# Long‐term risk of acute pancreatitis in patients with celiac disease: A nationwide population‐based cohort study

**DOI:** 10.1111/joim.70074

**Published:** 2026-02-13

**Authors:** Jialu Yao, Jiangwei Sun, Fahim Ebrahimi, David Bergman, Peter H. R. Green, Benjamin Lebwohl, Daniel A. Leffler, David S. Sanders, Björn Lindkvist, Miroslav Vujasinovic, Jonas F. Ludvigsson

**Affiliations:** ^1^ Department of Medical Epidemiology and Biostatistics Karolinska Institutet Stockholm Sweden; ^2^ Department of Gastroenterology and Hepatology University Digestive Health Care Center Basel—Clarunis Basel Switzerland; ^3^ Department of Medicine, Celiac Disease Center Columbia University College of Physicians and Surgeons New York New York USA; ^4^ Department of Epidemiology, Mailman School of Public Health Columbia University New York New York USA; ^5^ Celiac Center, Beth Israel Deaconess Medical Center Harvard Medical School Boston Massachusetts USA; ^6^ Chugai Pharmaceuticals USA Berkeley Heights New Jersey USA; ^7^ Academic Unit of Gastroenterology Sheffield Teaching Hospitals NHS Foundation Trust Sheffield UK; ^8^ School of Medicine and Population Health University of Sheffield Sheffield UK; ^9^ Department of Medicine Sahlgrenska University Hospital Gothenburg Sweden; ^10^ Department of Upper Abdominal Diseases Karolinska University Hospital Stockholm Sweden; ^11^ Department of Medicine, Huddinge Karolinska Institutet Stockholm Sweden; ^12^ Department of Pediatrics Örebro University Hospital Örebro Sweden

**Keywords:** acute pancreatitis, celiac disease, cohort, nationwide

## Abstract

**Background:**

Large‐scale studies on the association between celiac disease (CeD) and acute pancreatitis (AP) are scarce.

**Objectives:**

To investigate the long‐term risks of incident and recurrent AP in patients with CeD.

**Methods:**

Through the Swedish nationwide histopathology cohort Epidemiology Strengthened by Histopathology Reports in Sweden, we collected data on biopsy‐confirmed CeD diagnosed between 1969 and 2023 (*n* = 57,221) and matched them with general population reference individuals (*n* = 279,126) by birth year, sex, calendar year, and county. Cox regression estimated average adjusted hazard ratios (aHRs) for incident and recurrent AP over time, whereas flexible parametric survival models assessed time‐varying incident risks.

**Results:**

During a median follow‐up of 15.5 years, incident AP occurred in 549 patients with CeD (incidence rate [IR]: 58.7/100,000 person‐years), and 1732 reference individuals (IR: 37.8). The multivariable‐adjusted hazard for *incident* AP was consistently increased in patients with CeD compared with reference individuals (aHR = 1.42 [95% confidence intervals {CI}: 1.28–1.58]), resulting in one extra incident AP event per 185 CeD patients during the first 25 years after diagnosis. Increased incident risks were observed for gallstone‐ and non‐gallstone‐related AP, and severe AP, but not alcohol‐related AP. Conversely, in study participants who had survived a first AP episode, CeD was not associated with an increased risk for *recurrent* AP (aHR = 0.85 [0.67–1.08]). Sensitivity analyses, including a sibling comparison, confirmed the main findings.

**Conclusion:**

CeD is linked to a moderately increased long‐term risk of incident AP, but not to recurrent AP after the first episode. Clinicians should be aware of this increased risk and counsel patients with CeD on AP risk factors.

## Graphical abstract

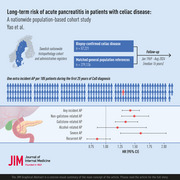

**Abbreviations**
AP: acute pancreatitis
ATC: Anatomical Therapeutic Chemical
CeD: celiac disease
CI: confidence interval
ESPRESSO: Epidemiology Strengthened by Histopathology Reports in Sweden
ICD: International Classification of Diseases
LISA: the Swedish Longitudinal Integrated Database for Health Insurance and Labour Market Studies
NPR: National Patient Register
PPV: positive predictive value
SNOMED: Systematized Nomenclature of Medicine
TPR: Total Population Register
VA: villus atrophy

## Introduction

Celiac disease (CeD) is an autoimmune condition that affects genetically susceptible individuals, with a global prevalence of around 1% [1, 2]. Individuals with CeD develop small intestinal villus atrophy (VA) and inflammation upon gluten exposure and therefore require lifelong adherence to a gluten‐free diet [1]. Owing to shared genetic predispositions and immunological pathways, as well as the psychosocial and nutritional implications associated with disease management, CeD is linked to a spectrum of complications within and beyond the small intestine [1]. Acute pancreatitis (AP) is a potentially lethal condition characterized by sudden inflammation of the pancreatic parenchyma and peripancreatic tissues, most commonly presenting with severe upper abdominal pain [3]. Gallstones and alcohol are the leading causes of AP, whereas autoimmune conditions, gene aberrations, and medications are also among its risk factors [3]. Following a first AP episode, one meta‐analysis has summarized that about 22% of patients will experience recurrent AP, whereas 10% will develop chronic pancreatitis [4].

To date, only six (including one abstract) multi‐centre or population‐based studies have examined the association between CeD and AP (summarized in Table S1) [5, 6, 7, 8, 9, 10]. Although a positive association has been consistently reported, these studies have several limitations, including outdated data (i.e., follow‐up ended some 10−20 years ago) [7, 8, 9], short follow‐up (i.e., median 5 and 10 years for *any pancreatitis* in the two studies with available information) [8, 9] and selection of reference individuals from healthcare databases that may have led to risk underestimation [5, 6, 7, 10]. Moreover, existing studies have not investigated the risk of recurrent AP in patients with CeD, despite a notable co‐occurrence in a case series study conducted almost 30 years ago [11].

Using up‐to‐date cohort data capturing diagnoses through 2023 for CeD and August 2024 for AP, we conducted a population‐based cohort study to investigate the long‐term risk of AP in patients with CeD. We hypothesized that CeD is associated with an increased risk for incident AP and its recurrence. In addition, we also assessed whether persistent VA in CeD conferred a higher risk for AP compared to mucosal healing.

## Methods

### Data sources

This study was based on the ESPRESSO (Epidemiology Strengthened by Histopathology Reports in Sweden) cohort, which collects gastrointestinal histopathology reports from all 28 pathology departments in Sweden from 1965 and updated through 2023 [12]. The ESPRESSO cohort contains information on the date, anatomic location, and morphology (by the Swedish version of the Systematized Nomenclature of Medicine system) of the histopathology reports [12].

Individuals from ESPRESSO are linked with nationwide registers through the unique personal identity number [13]. The Swedish National Patient Register (NPR) contains data on diagnoses, procedures and deaths from inpatient care (commenced in 1964 and became nationwide since 1987) and specialized outpatient care (since 2001) [14]. A 2011 review summarized that inpatient records in the NPR were correct for 85%−95% of diagnoses and 90%−97% of procedures [15]; a later validation estimated median positive predictive values (PPVs) of 84% for diagnoses and 97% for procedures across inpatient and outpatient records [14]. Both primary and secondary diagnoses were considered for relevant outcomes and disease history. We also used data from the Swedish Cancer Register (commenced in 1958, covering >96% incident cancer cases) and the Prescribed Drug Register (contains data on prescribed medications since 1 July 2005) [16, 17].

### Patients with CeD and comparison groups

Patients with biopsy‐confirmed CeD between 1969 and 2023 were identified from ESPRESSO, using VA (Marsh 3) in the duodenum or jejunum as our CeD definition (see Table S2 for relevant codes) [12]. This algorithm has a PPV of ≥95% [18, 19]. For patients with CeD, the index date was the date of their first biopsy, indicating duodenal/jejunal VA (i.e., the date of CeD diagnosis).

We matched each patient with CeD with up to five general population reference individuals from the Total Population Register (TPR) by sex, birth year, year of the index date and county of residence [13]. For reference individuals, the index date was the date of them being matched.

Exclusion criteria are detailed in Table S3. Briefly, we excluded individuals with diagnoses or prescriptions indicating any preceding pancreatic condition or cystic fibrosis, or those with procedures in the pancreas or cholecystectomy before the index date.

### Persistent VA and mucosal healing

Among included patients with CeD, we identified those who had a follow‐up biopsy between 6 months and 5 years after the index date. These patients were defined as having persistent VA (Marsh 3) or mucosal healing (Marsh 0−2) in the follow‐up biopsy. The index date for these patients was the date of their follow‐up biopsy, and the same exclusions as for the main comparison were applied.

### Follow‐up and outcome ascertainment

The primary outcome was any incident AP identified from the NPR (previous validation showed that 83% of AP diagnoses were definitive and 15% were probable [20]). Secondary outcomes included AP of different aetiologies (including gallstone‐related, non‐gallstone‐related and alcohol‐related AP), severe AP, and recurrent AP (defined in Fig. S1 and Table S4a).

Follow‐up started from the index date and ended at the date when criteria for each outcome were fulfilled, or the earliest occurrence of (a) an incident diagnosis of pancreatic cancer or chronic pancreatitis (to avoid misclassifying acute flare‐up as AP), (b) death, (c) emigration or (d) the study end (31 August 2024). Reference individuals were censored and reclassified into the patient group at the date of CeD diagnosis. When investigating the risk of non‐gallstone‐related AP, individuals were additionally censored with incident gallstone‐related diagnoses or procedures.

In the main analysis, an AP episode began with admission to AP‐related specialized care and ended at discharge (i.e., presumed date of remission). Individuals were at risk for a subsequent episode starting 90 days after discharge, following the AGA [the American Gastroenterological Association] guideline [21]. Gallstone‐related AP was defined as (a) any incident diagnosis of biliary pancreatitis or (b) any gallstone‐related diagnosis or procedure ever before incident AP or <90 days after discharge. Incident AP records that did not meet these criteria at 90 days of discharge were defined as non‐gallstone‐related. Alcohol‐related AP was defined as non‐gallstone‐related, non‐drug‐induced AP preceded by diagnostic or medication codes indicating heavy alcohol consumption [22].

Severe AP was a composite outcome comprising (a) ≥14 days of hospitalization for AP, (b) receipt of a diagnostic or procedural code implying a complicated episode, (c) or all‐cause death <90 days of discharge for AP. Recurrent AP was treated as a recurrent event. Among individuals who had survived the first episode, subsequent episodes of AP were identified iteratively if they occurred ≥90 days after discharge from the most recent episode, up to the occurrence of any censoring events. Records occurring <90 days were considered part of the same episode [21].

### Covariates

Several covariates, in addition to matching variables, were adjusted for. **First,** educational attainment (0−9, 10−12, ≥13 years, or ‘missing’), a proxy for socioeconomic status, was ascertained from LISA (the Swedish Longitudinal Integrated Database for Health Insurance and Labour Market Studies) (education data were correct for 85% of individuals in LISA) [23]. For those <22 years at the index date, we took the highest educational attainment of the individual or their parents. **Second,** country of birth (Nordics [Sweden, Denmark, Finland, Norway and Iceland] or others) was collected from the TPR [24]. **Third,** number of specialized healthcare visits 6−24 months before the index date (0, 1, 2−3 or ≥4), a proxy indicating healthcare‐seeking frequency, was retrieved from the NPR. **Lastly,** we included a dichotomous covariate for the presence of any autoimmune disease before the index date (see Table S4a for related codes).

### Statistical analyses

Time since the index date was the underlying time scale. We reported absolute risks of the investigated outcomes with both unadjusted incidence rates (IRs) and their between‐group differences, as well as the standardized (covariate‐adjusted) cumulative incidence [25].

Adjusted hazard ratios (aHRs) were estimated with Cox proportional hazards models for relative risks. The proportional hazards assumption was tested using Schoenfeld residuals‐based test, with its violation observed in some models. Therefore, the resulting HR was a summary of averaged HRs across follow‐up time [26]. To capture the time‐varying effect of CeD, we also presented the standardized cumulative incidence and adjusted HRs over follow‐up time, using flexible parametric survival models [25, 27]. Risk estimates were presented with 95% confidence intervals (CIs).

Adjusted HRs and standardized cumulative incidence were estimated using two models. Model 1 adjusted for the matching variables, including sex, birth year, year of the index date and county of residence. Model 2 further adjusted for educational attainment, country of birth, number of specialized healthcare visits and autoimmune disease history. When comparing between patients with VA and those with mucosal healing, the duration of CeD diagnosis at the date of follow‐up biopsy was additionally adjusted for.

When investigating the risk of any recurrent AP, we first described its frequency among individuals who were at risk after the first AP episode. Then, a Prentice‐Williams‐Peterson‐counting process data structure was applied for its relative risk while accounting for the dependency between recurrent episodes (i.e., individuals were at risk of a new AP episode only after a previous attack) and the changing baseline hazards [3, 28]. We only considered the first three recurrence episodes for statistical power [28]. Within‐individual correlations were corrected with the clustered sandwich estimators [27]. In this data structure, follow‐up restarted at 90 days after discharge from the most recent AP, and covariates for the survival model were updated [27, 28].

### Subgroup and sensitivity analyses

We estimated the risks of incident AP by age at index date (<18, 18 to <40, 40 to <60, ≥60 years), sex, country of birth, calendar period at index date (1969−1989, 1990−2001, 2002−2011 and 2012−2023), educational attainment, and history of metabolic‐related diseases (i.e., hypertension, diabetes mellitus, obesity, dyslipidaemia), autoimmune diseases, heavy alcohol consumption and chronic obstructive pulmonary disease diagnosed ≥40 years (as a proxy for heavy smoking [29], see Table S4a for related codes).

The following sensitivity analyses were conducted. **First,** we applied alternative time intervals for certain secondary outcomes. This included shortening the risk intervals for gallstone‐ or non‐gallstone‐related AP and for all‐cause death (severe AP component) from 90 to 30 days after discharge, and defining the date of remission as the last discharge date in a series of AP‐related specialized care occurring <90 days apart. Results from this sensitivity analysis were presented with corresponding secondary outcomes.

**Second,** to account for possible intra‐familial confounding [2, 3], we conducted a sibling comparison. CeD‐free full siblings of patients with CeD were identified from the Swedish Multi‐Generation Register (a TPR component) as a comparison group and followed from the date of their sibling's CeD diagnosis [24]. A family identifier was included in addition to the multivariable model covariates in the main analysis. **Third,** we used logistic regression to investigate whether CeD was also positively associated with prior AP. **Fourth,** we restricted the analyses to those with educational attainment data (after 1990) to further address its potential confounding [23]. **Fifth,** we excluded the first follow‐up year to ascertain the temporal relationship between CeD and AP. **Sixth,** to eliminate the potential impact of the COVID‐19 pandemic on the risk of AP in the general population, we changed the study end to 31 December 2019 [30]. **Finally,** some medications for refractory CeD may be positively linked to AP [31, 32, 33]. To rule out their potential impact, we restricted our analyses to individuals who had an index date from January 2006 onward (i.e., 6 months after the start of the Prescribed Drug Register to exclude prevalent users) and were naïve to any or each of steroids, mesalamine and immunosuppressants at the index date. Individuals were also censored when receiving the listed prescriptions (see Table S4b for relevant codes) [17].

Data analyses and visualizations were conducted in Stata (version 18.0; StataCorp LP) and R version 4.3.1. A two‐sided *p *≤ 0.05 was considered statistically significant.

### Ethics

This study was approved by the Stockholm Ethics Review Board (2014/1287‐31/4, 2018/972‐32 and 2022‐05774‐02). Individual informed consent was waived as the study was register‐based.

## Results

We included 57,221 patients diagnosed with biopsy‐confirmed CeD between 1969 and 2023 and 279,126 matched reference individuals from the general population (see Fig. S2 for individual selection). Among patients with CeD, the median age at diagnosis was 28.4 years, 62.3% were female, and 30.8% were diagnosed since 2012. Other characteristics of patients with CeD and reference individuals, including educational attainment, number of healthcare visits and disease history, were summarized in Table 1. Some 34.4% of patients were followed for ≥20 years.

**Table 1:** Characteristics of patients with CeD and their matched reference individuals, n (%).

	Patients with CeD	References
*N*	57,221	279,126
Age at index date, years^a^		
Mean ± SD	32.2 ± 24.7	31.5 ± 24.3
Median (IQR)	28.4 (9.6–52.3)	27.4 (9.2–51.1)
<18	22,005 (38.5%)	109,844 (39.4%)
18 to <40	13,731 (24.0%)	67,827 (24.3%)
40 to <60	11,237 (19.6%)	54,401 (19.5%)
≥60	10,248 (17.9%)	47,054 (16.9%)
Female	35,629 (62.3%)	173,907 (62.3%)
Born in Nordic countries^b^	54,236 (94.8%)	251,139 (90.0%)
Calendar period at index date		
1969–1989	3302 (5.8%)	16,160 (5.8%)
1990–2001	16,380 (28.6%)	79,703 (28.6%)
2002–2011	19,942 (34.9%)	97,326 (34.9%)
2012–2023	17,597 (30.8%)	85,937 (30.8%)
Educational attainment, years		
0–9	7701 (13.5%)	40,284 (14.4%)
10–12	22,499 (39.3%)	111,104 (39.8%)
≥13	24,262 (42.4%)	113,706 (40.7%)
Missing	2759 (4.8%)	14,032 (5.0%)
Number of healthcare visits^c^		
0	36,188 (63.2%)	207,031 (74.2%)
1	8778 (15.3%)	35,023 (12.5%)
2–3	6480 (11.3%)	22,051 (7.9%)
≥4	5775 (10.1%)	15,021 (5.4%)
History of metabolic‐related diseases^d^	7854 (13.7%)	26,505 (9.5%)
Hypertension	4679 (8.2%)	19,877 (7.1%)
Diabetes	3214 (5.6%)	6638 (2.4%)
Obesity	484 (0.8%)	2876 (1.0%)
Dyslipidaemia	2633 (4.6%)	10,928 (3.9%)
History of autoimmune diseases^d^	6241 (10.9%)	8233 (2.9%)
History of heavy alcohol consumption^d^	922 (1.6%)	5037 (1.8%)
History of COPD ≥40 years^c^, ^d^	371 (0.6%)	1393 (0.5%)
Follow‐up time, years		
Mean ± SD	16.3 ± 9.6	16.4 ± 9.6
Median (IQR)	15.5 (8.5–23.0)	15.5 (8.5–23.2)
0–0.9	1026 (1.8%)	3856 (1.4%)
1–4.9	6585 (11.5%)	32,996 (11.8%)
5–9.9	9798 (17.1%)	47,597 (17.1%)
10–19.9	20,129 (35.2%)	98,087 (35.1%)
≥20	19,683 (34.4%)	96,590 (34.6%)

Abbreviations: CeD, celiac disease; COPD, chronic obstructive pulmonary disease; IQR, interquartile range; SD, standard deviation.

^a^ Date of CeD‐indicative biopsy for patients with CeD and date of selection for reference individuals.

^b^ Nordic countries: Sweden, Denmark, Finland, Norway and Iceland.

^c^ Between 2 years and 6 months before the date of index date.

^d^ Codes for disease histories are listed in Table S4.

^e^ Proxy for heavy smoking [29].

### Primary outcome: any incident AP

**Fig. 1:**
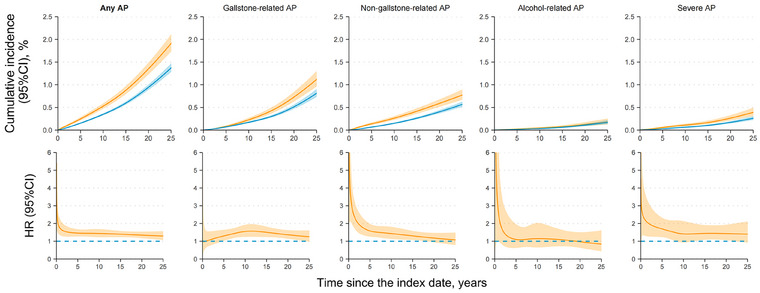
Standardized cumulative incidence (top) and hazard ratio (bottom) for acute pancreatitis in patients with celiac disease compared with their matched reference individuals (in lines), both with 95% confidence interval (in ribbons). The hazard ratio and standardized cumulative incidence were estimated with the flexible parametric survival model while being adjusted for covariates in model 2. CI, confidence interval; AP, acute pancreatitis; HR, hazard ratio.

During follow‐up (15.5 years in median), AP developed in 549 patients with CeD (IR: 58.7 per 100,000 person‐years) and 1732 reference individuals (IR: 37.8). This corresponded to an average HR of 1.42 [95%CI: 1.28–1.58] in patients with CeD compared with reference individuals after multivariable adjustment (Table 2). The HR for any AP was highest immediately after CeD diagnosis, fell to 1.5 around Year 3 but stayed above 1.3 through Year 25 after diagnosis, resulting in a 25‐year difference in standardized cumulative incidence of 0.54% (0.36%–0.72%) (Fig. 1 and Table S5).

**Table 2:** Incident AP in patients with CeD and their matched reference individuals.

	No. of events (IR, per 100,000 person‐years)		IR difference (95%CI), per 100,000 person‐years	HR (95%CI)	
	Patients	References		Model 1^a^	Model 2^b^
Primary outcome					
Any AP^c^	549 (58.7)	1732 (37.8)	20.9 (15.7–26.1)	1.54 (1.39–1.70)	1.42 (1.28–1.58)
Secondary outcomes					
Gallstone‐related AP	260 (27.8)	898 (19.6)	8.2 (4.6–11.8)	1.40 (1.21–1.61)	1.34 (1.16–1.55)
Non‐gallstone‐related AP	255 (27.3)	746 (16.3)	11.0 (7.4–14.5)	1.70 (1.47–1.97)	1.49 (1.27–1.74)
Alcohol‐related AP	47 (5.0)	173 (3.8)	1.3 (−0.3 to 2.8)	1.32 (0.95–1.83)	1.20 (0.85–1.70)
Severe AP	107 (11.4)	298 (6.5)	4.9 (2.6–7.2)	1.78 (1.41–2.24)	1.60 (1.25–2.04)
Recurrent AP^d^, among 507 patients with CeD and 1588 reference individuals who were at risk after the first episode	99 (2632.7)	345 (2910.1)	−277.4 (−880.1 to 325.3)	0.88 (0.70–1.11)	0.85 (0.67–1.08)

Abbreviations: AP, acute pancreatitis; CeD, celiac disease; CI, confidence interval; HR, hazard ratio; IR, incidence rate.

^a^ Model 1: conditioned on the matching variables (birth year, sex, county of residence and calendar year of index date).

^b^ Model 2: Model 1 and further adjusted for country of birth, educational attainment, number of healthcare visits between 2 years and 6 months before the index date, and the history of autoimmune diseases.

^c^ Event numbers for gallstone‐related and non‐gallstone‐related AP did not add up to that for any AP. There are two potential reasons: First, individuals with incident non‐gallstone‐related AP were still at risk for later gallstone‐related AP; second, some individuals, although still at risk for both types of AP (e.g., an individuals with incident idiopathic AP [ICD‐10: K850] but no history of gallstone‐related diagnoses or procedures), may be censored due to incident diagnosis of pancreatic cancer or chronic pancreatitis, death, emigration, study end or incident diagnosis of celiac disease (for reference individuals) within 90 days of charge from incident AP diagnosis.

^d^ Restricted to the first three recurrence episodes for statistical power.

Patients with CeD had increased absolute and relative risks for any incident AP across adults, both sexes and those diagnosed after 1990 (Table S6). Higher HR estimates were observed in individuals with low (0−9 years) education (aHR = 1.88 [1.47–2.41], *P_interaction_* = 0.03) and those with prior diagnoses or medications indicating heavy alcohol consumption (aHR = 3.79 [1.75–8.23], *P_interaction_* < 0.001). The associations persisted in individuals with no history of metabolic‐related diseases or autoimmune diseases.

### Secondary outcomes: incident AP by aetiology and severe AP

In patients with CeD, absolute and relative risks were significantly elevated for non‐gallstone‐related AP (IR: 27.3 vs. 16.3 per 100,000 person‐years; aHR = 1.49 [1.27–1.74]) and gallstone‐related AP (IR: 27.8 vs. 19.6; aHR = 1.34 [1.16–1.55]), but not for alcohol‐related AP (IR: 5.0 vs. 3.8; aHR = 1.20 [0.85–1.70], Table 2). The temporal patterns of HR differed by aetiology: For non‐gallstone‐related AP, the highest HR emerged instantly after diagnosis but steadily decreased, with the hazard increase becoming statistically non‐significant from around Years 17−19 post CeD diagnosis. Conversely, the increased hazard for gallstone‐related AP rose to a significant level from Year 5, peaked around Years 10−11, and remained significant until Year 25. The HR estimate for non‐gallstone‐related AP was surpassed by gallstone‐related AP from around Year 8 onward (Fig. 1).

CeD was associated with a 60% increased hazard for severe AP (95%CI: 1.25–2.04; composed of hospitalization ≥14 days or with severe complications, or mortality <90 days of discharge), although the absolute risk excess was low (IR: 11.4 vs. 6.5). Subgroup analysis results for AP of different aetiologies and severe AP were shown in Table S7. The associations with these secondary outcomes, except for alcohol‐related AP, were strongest among individuals with low education, although *P*_interaction_ did not reach statistical significance. History of heavy alcohol consumption significantly strengthened the associations of CeD with non‐gallstone‐related AP, alcohol‐related AP and severe AP.

### Secondary outcome: recurrent AP

Up to the study end, a total of 104 AP episodes recurred in patients with CeD (*n* = 507) and 375 in reference individuals (*n* = 1588) who were at risk for recurrent AP (Table S8). These patients with CeD had a median age of 62.2 years and were 55.2% female. Among them, the median follow‐up for the *second* AP episode was 4.5 years. Metabolic‐related diseases were less common in patients with CeD compared to reference individuals (51.9% vs. 53.7%). When following up for all recurrent AP episodes, the frequency of censoring events in patients with CeD was 3.4% for chronic pancreatitis (vs. 3.3% in reference individuals), 1.4% for pancreatic cancer (vs. 0.4% in reference individuals), and 30.0% for death (vs. 26.1% in reference individuals). The risk for any recurrent AP (based on the first three recurrence episodes) was *not* higher in patients with CeD in either absolute or relative terms (IR: 2632.7 vs. 2910.1 per 100,000 person‐years; aHR = 0.85 [0.67–1.08], Table 1).

The associations of CeD with AP of different aetiologies and severe AP, as well as with recurrent AP, were not markedly affected by varied time intervals in their definitions (see Fig. S3 for graphic definitions and results in Table S9).

### Impact of persistent VA on AP risk

We identified 15,965 patients with CeD who underwent a follow‐up biopsy between 6 months and 5 years after diagnosis. Among them, 4099 patients (25.7%) had persistent VA (Table S10). Compared with patients showing mucosal healing, those with persistent VA were about 19 years older (in median), more often male, and had a lower educational attainment. Patients with persistent VA showed a higher absolute risk for any AP (IR: 88.1 vs. 57.1 per 100,000 person‐years), but no excess in its hazard (aHR = 0.92 [0.66–1.27]) after multivariable adjustment. The two patient groups did not significantly differ in absolute or relative risks for AP of any aetiology or severe AP (Table S11).

### Sensitivity analyses

We compared 40,256 patients with CeD with their 69,439 CeD‐free full siblings (baseline characteristics in Table S12). Consistent with the general population comparison, patients with CeD had increased hazards for any AP (aHR = 1.30 [1.10–1.53]; specifically for non‐gallstone‐related AP: aHR = 1.32 [1.03–1.70]) and severe AP (aHR = 1.85 [1.16–2.94], and the association between CeD and alcohol‐related AP remained non‐significant (aHR = 1.21 [0.67–2.19]). However, the hazard for gallstone‐related AP was no longer significantly elevated in patients with CeD in the sibling comparison (aHR = 1.19 [0.94–1.50]; Table S13).

The odds for any AP, non‐gallstone‐related AP and severe AP were also increased before CeD diagnosis (Table S14).

Restricting analyses to individuals with available educational attainment data, excluding the first follow‐up year, and ending follow‐up before the COVID‐19 pandemic yielded similar results to the main analysis. In analyses restricted to individuals naïve to any or each of steroids, mesalamine and immunosuppressants (index date 2006−2023), the association between CeD and any AP was attenuated (Table S15). Hazard ratio estimates ranged between 1.16 and 1.23, lower than those observed in patients diagnosed from 2006 onward (see Table S15 footnotes), and the association became non‐significant in some analyses. CeD also showed reduced associations with AP of all investigated aetiologies and with severe AP (Table S15 footnotes). Only the positive association with non‐gallstone‐related AP remained significant.

## Discussion

This Swedish nationwide cohort study found a 42% increase in average relative risk for any incident AP in patients with biopsy‐confirmed CeD. The HR estimate plateaued between 1.3 and 1.5 from the third year and onward after CeD diagnosis, leading to one extra event of incident AP per 185 patients during the first 25 years. The association was even stronger in patients with low educational attainment and those with a history of heavy alcohol consumption.

The positive association remained in sensitivity analyses that accounted for potential residual confounding from shared familial risk factors and educational attainment, as well as risk inflation due to coincidental detection of the two conditions when investigating upper abdominal pain or increased diagnostic work‐ups at the time of CeD diagnosis (via exclusion of the first follow‐up year). However, the association with any AP was notably attenuated in individuals naïve to potentially AP‐inducing medications for refractory CeD, including steroids, mesalamine and immunosuppressants [31, 32, 33]. Research accounting for indication bias is needed to disentangle these medications’ influence in the CeD−AP association.

The persistent risk elevation for any AP was primarily driven by non‐gallstone‐related AP during the initial follow‐up (aHR estimate: 1.49), with gallstone‐related AP becoming the main driver from around Year 8 onward (aHR estimate: 1.34). This shift, reflecting varied temporal patterns of these two outcomes (Fig. 1), may be explained by differential risk patterns among patients diagnosed <18 years. As paediatric patients age during follow‐up, the relative risk for gallstone‐related AP is expected to increase, whereas that for non‐gallstone‐related AP may decrease (Table S7). In contrast, no significant association with alcohol‐related AP was observed throughout follow‐up. Patients with CeD also had a higher relative risk of experiencing a severe attack during the first AP episode (aHR estimate: 1.60), but no increased risk for subsequent recurrent AP (aHR estimate: 0.85). In addition, mucosal healing during follow‐up did not protect patients from any incident AP (persistent VA vs. mucosal healing, aHR estimate: 0.92).

### Comparison with earlier literature

We are aware of four studies on incident AP following a CeD diagnosis (see Table S1) [5, 6, 8, 9]. Consistent with earlier findings, we observed a heightened risk for any AP in patients with CeD [5, 6, 9] and a stronger association with non‐gallstone‐related AP compared to that related to gallstone [5, 6, 8]. However, relative risk estimates in this study were lower for two main potential reasons. **First,** the longer follow‐up gave less inflated risk estimates as more individuals contributed person‐years beyond the peak HR (Fig. 1). **Second,** the additional exclusion of individuals with pancreatic cancer and chronic pancreatitis before and during follow‐up may have also reduced the risk estimates, given the two‐ and three‐fold increased risks of these two conditions in patients with CeD [8, 34].

In this study, among individuals who survived their first AP episode and remained free from chronic pancreatitis or pancreatic cancer within 90 days after discharge, the risk for AP recurrence was not elevated in patients with CeD compared to reference individuals. This was opposed to the positive association suggested by Patel et al., who observed 10 recurrent AP out of 12 CeD patients investigated for Sphincter of Oddi dysfunction [11]. The same study has postulated that papillary stenosis may predispose CeD to recurrent AP [11]. However, that study had limitations inherent to its case series design, including selection bias (i.e., observed patients were referred to a single gastroenterology centre with a higher risk of an AP history) and lack of a comparison group. The null association in this study may have several explanations. **First,** despite possible pathological susceptibility, patients with CeD may have received better preventive measures against recurrent AP, including a more frequent healthcare contact, particularly with the gastroenterologists after a remission of the first episode. Relatedly, physicians may be more cautious when prescribing potentially AP‐inducing medications, with better knowledge of these patients’ medical histories. **Second,** the lower prevalence of metabolic risk factors, such as obesity and diabetes in patients with CeD (Table S8), may have compensated the risk for recurrent AP [3]. Nevertheless, selection bias is possible, as patients with CeD had more frequent censoring due to incident pancreatic cancer or death, leaving a healthier subset at risk for recurrence. In addition, in the selected population that survived the first AP episode, immune dysfunction may have contributed disproportionally to the initial event among patients with CeD, whereas reference individuals were more likely to have stronger recurrence‐prone aetiologies, such as alcohol abuse and smoking [3].

### Potential mechanisms

CeD may contribute to both the initiation of AP (i.e., acinar cell damage) and its subsequent inflammation, although we could only speculate about underlying mechanisms because they are beyond our study scope. Patients with CeD may be predisposed to gallstone formation due to reduced gallbladder motility, potentially resulting from dysregulated cholecystokinin signalling in VA [35, 36]. In addition, a case‐series study noted increased hepatic cholesterol secretion in patients with CeD, and resulting supersaturation of primary bile acids may exacerbate the risk for gallstone‐induced acinar damage [35, 37].

CeD may also be linked to acinar damage by non‐gallstone‐related causes such as medications and infections. The AP‐inducing role of steroids, mesalamine and immunosuppressants was implicated by the risk attenuation in medication‐naïve analyses (Table S15). However, there is potential influence of indication bias as patients naïve to these medications may have had a lower prevalence of refractory CeD or comorbidities, such as inflammatory bowel disease (also treated with these medications [31, 32], and independently associated with both CeD and AP [38, 39]). Although we cannot rule out this possibility, research has specifically linked the CeD‐predisposing HLA variant DQ2.2 to immunosuppressant‐induced AP [40, 41], suggesting a genetically mediated association. Infections may also play a role. Although viral, bacterial and protozoal infections are rare causes of AP [3], the higher risk of severe infections in CeD supports their potential contribution [42].

Inflammation that follows acinar damage can be perpetuated in the presence of dysfunctional intestinal barrier and aberrant immune responses in CeD. Damaged acinar cells trigger innate immune responses that lead to increased intestinal permeability, translocation of commensal bacteria, and macrophage‐regulated adaptive immune response [43, 44]. This process may be amplified in CeD due to pre‐existing intestinal barrier impairment [45]. CeD also shares pathological features with AP, including increased pro‐inflammatory cytokines such as type I interferon and interleukin‐6, which can sustain pancreatic inflammation [41, 43]. Moreover, both bacterial translocation and interleukin‐6 may be involved in systemic inflammation and organ failure that complicate AP, leading to CeD patients’ susceptibility to a severe episode [44, 45, 46].

Notably, although prolonged impairment in cholecystokinin signalling and intestinal barrier function may elevate the AP risk [35, 41], this study observed no excess risk in patients with persistent VA compared to those showing mucosal healing in the follow‐up biopsy. More severe malabsorption in the persistent VA group, potentially leading to a lower prevalence of metabolic risk factors (e.g., hypercalcemia and obesity) [1, 3], may offset the risk for AP. In addition, the first follow‐up biopsy may not perfectly predict long‐term mucosal status, and subsequent changes in these two groups may also explain the lack of significant difference [47].

### Strengths and limitations

The population‐based design of this study was enabled by two key factors. **First,** we used a nationwide histopathology cohort and a highly accurate algorithm (PPV≥95%) to identify CeD [18, 19], reducing selection bias. **Second,** Sweden's universally accessible healthcare and nationwide registers provided prospectively collected, high‐quality demographic and healthcare data [13, 48]. These enabled a virtually complete follow‐up of over 57,000 patients with biopsy‐confirmed CeD. The sample size and comprehensiveness provided high statistical power and information to explore AP risk by aetiology, severity and recurrence, and across informative subgroups. The long follow‐up (with over 34% of patients followed for ≥20 years) was especially valuable, given the large age gap between CeD diagnosis (i.e., median 28 years, Table 1) and AP onset in the general population (i.e., median 62 years) [49]. Information bias was further mitigated by the high validity of International Classification of Diseases codes used to identify AP (PPV = 98% for definitive and probable cases) [20].

There are also limitations. **First,** the serology‐based approach became an option for diagnosing CeD in Sweden since 2012 for children and 2020 for adults [50, 51]. Therefore, despite our nationwide coverage of biopsy‐confirmed CeD, we have missed cases that were diagnosed solely via serology. Although HR estimates before and after 2012 were largely similar (Tables S6 and S7), future research incorporating serology‐confirmed cases with longer follow‐up in the post‐serology era is warranted. **Second,** we could not rule out undiagnosed CeD cases in reference individuals; their presence may have diluted the association with AP. **Third,** although diagnoses of AP in the Swedish NPR have a high overall PPV [20], their underlying aetiologies are often not established during the initial admission, with 65%−71% (=125/192 to 110/156) of AP records during 2007 coded as ‘AP, unspecified’ [20]. To improve sensitivity for detecting gallstone‐related AP, we introduced a 90‐day post‐discharge risk time. This approach has been used in several peer‐reviewed studies [49, 52], with the only difference being that their additional risk time was counted from admission. Despite the nuance, the validity of our adapted definition was supported by comparable IRs in the reference individuals with those reported in the general population (see detailed comparison in Table S7 footnotes) [49]. Nevertheless, the misclassification of AP aetiologies could not be excluded. For example, some gallstone‐related diagnoses or procedures (part of the algorithm to identify gallstone‐related AP) may have been missed before the NPR became nationwide (in 1987) or had outpatient (in 2001) coverage. If such under‐ascertainment were more common among CeD patients given their proposed pathological susceptibility to gallstones [35], the association with gallstone‐related AP would have been underestimated. **Fourth,** the Prescribed Drug Register does not contain treatment indications, impeding attribution of medication use [17]. Further research is needed to disentangle the effect of medications for refractory CeD from the condition per se in the observed association.

**Fifth,** we lacked detailed data on lifestyle and metabolic risk factors for AP, such as alcohol abuse, smoking and obesity [3]. Although we used diagnostic or medication records as proxies, under‐reporting remains. **Sixth,** the impact of a gluten‐free diet could not be directly determined in the absence of the dietary data. In this study, mucosal healing in the follow‐up biopsy was used as a proxy for dietary adherence [53]. However, this proxy has limitations as it may not reflect long‐term adherence [54], and mucosal healing is possible, albeit rarely, in cases with rather poor adherence [47]. **Seventh,** over 90% of included individuals were born in the Nordic countries, where the population has a high genetic susceptibility for CeD but the underlying Caucasian predominance implies a low ethnic predisposition for AP [2, 3]. Caution should therefore be placed when extrapolating the relative and absolute risk estimates to other populations, particularly those with different ethnic compositions. **Finally,** mechanisms linking CeD to AP could not be ascertained in this observational study, and the observed association should not be interpreted as causal.

### Implications

The rising global incidence of AP urges measures from its primary prevention to mitigation of its adverse impact [3]. In this study, we demonstrate that patients with CeD would benefit more from lifestyle modifications and clinical prudence than the general population in reducing AP risk. For patients with CeD, dietary counselling to ensure a nutritionally balanced gluten‐free diet and cautious use of AP‐inducing medications are particularly relevant. In addition, the potential symptom overlap between the two conditions, such as acute upper abdominal pain, underscores clinical vigilance to facilitate early detection of AP and to prevent its severe progression as well as subsequent complications.

In conclusion, we found a moderate (aHR 1.42), but persistent (>25 years), increased risk for incident AP in patients with CeD. However, the risk of recurrent AP after a first episode was not elevated in CeD. Clinicians treating patients with CeD should be aware of the heightened incident risk of AP and counsel patients on its risk factors.

## Author contributions

Study concept and design: Jialu Yao, Jiangwei Sun, Jonas F. Ludvigsson, Fahim Ebrahimi, David Bergman, Peter H. R. Green, Daniel A. Leffler, David S. Sanders, Benjamin Lebwohl, Björn Lindkvist and Miroslav Vujasinovic. Acquisition of data: Jonas F. Ludvigsson. Drafting of the manuscript: Jialu Yao and Jonas F. Ludvigsson. Interpretation of data and critical revision of the manuscript for important intellectual content: Jialu Yao, Jiangwei Sun, Jonas F. Ludvigsson, Fahim Ebrahimi, David Bergman, Peter H. R. Green, Daniel A. Leffler, David S. Sanders, Benjamin Lebwohl, Björn Lindkvist and Miroslav Vujasinovic. Statistical analysis: Jialu Yao. Funding acquisition: Jonas F. Ludvigsson. Administrative, technical, or material support: Jonas F. Ludvigsson. Guarantors: Jialu Yao and Jonas F. Ludvigsson have directly accessed and verified the underlying data reported in the manuscript and take responsibility for the integrity of the data, the accuracy of the data analysis, and the decision to submit the manuscript.

## Disclosure

The funders had no role in the design and conduct of the study; collection, management, analysis, and interpretation of the data; preparation, review or approval of the manuscript; and decision to submit the manuscript for publication.

## Conflicts of interest statement

All authors have completed the ICMJE uniform disclosure form and declare: Fahim Ebrahimi has served as an advisory board member for Boehringer Ingelheim. Daniel A. Leffler receives a salary as an employee of Chugai Pharmaceuticals and owns stock in this company. Jonas F. Ludvigsson has coordinated a study on behalf of the Swedish IBD quality register (SWIBREG). That study received funding from Janssen Corporation. Jonas F. Ludvigsson has also received financial support from MSD developing a paper reviewing national healthcare registers in China. Jonas F. Ludvigsson also has a research collaboration on celiac disease with Takeda. Björn Lindkvist has received speaker's honoraria from All About Meetings and Mediahuset. Miroslav Vujasinovic receives lecture fees from Abbott, Viatris, Nordmark Pharma, and Amgen. Miroslav Vujasinovic is also an advisory board member of Abbott and Amgen. The other authors report no disclosures relevant to the manuscript.

## Funding information

European Crohn's and Colitis Organisation (to Jiangwei Sun); the Swedish Society for Medical Research (to Jiangwei Sun, Grant number: PG‐23‐0315‐H‐02) and Takeda (to Jonas F. Ludvigsson)

## Supporting information




**Fig. S1**: *Graphic definition of investigated outcomes*.
**Fig. S2**: *Flow chart for study population selection*.
**Fig. S3**: *Alternative time intervals to define certain secondary outcomes*.
**Table S1**: *Multi‐centre or population‐based evidence on AP risk in CeD*.
**Table S2**: *SNOMED codes defining CeD*.
**Table S3**: *Definitions of exclusion criteria*.
**Table S4a**: *Definitions of outcomes and comorbidities*.
**Table S4b**: *Medications for refractory CeD that may be associated with AP*.
**Table S5**: *Cumulative incidence difference (95%CI) of incident AP during follow‐up in individuals with CeD, compared with their matched reference individuals*.
**Table S6**: *Subgroup analyses of any incident AP in patients with CeD and their matched reference individuals*.
**Table S7**: *Subgroup analyses of incident AP by different aetiologies and severe AP in patients with CeD and their matched reference individuals*.
**Table S8**: *Characteristics of patients with CeD and their matched reference individuals who were at risk for recurrent AP, n (%)*.
**Table S9**: *Sensitivity analyses of incident AP in patients with CeD and their matched reference individuals, by alternative time intervals to define certain secondary outcomes*.
**Table S10**: *Characteristics of CeD patients who had a follow‐up biopsy between 6 months and 5 years after diagnosis, n (%)*.
**Table S11**: *Incident AP in patients with CeD who had a follow‐up biopsy between 6 months and 5 years after diagnosis*.
**Table S12**: *Characteristics of patients with CeD and their full siblings, n (%)*.
**Table S13**: *Sensitivity analysis of incident AP in patients with CeD and their full siblings*.
**Table S14**: *Sensitivity analysis of prior AP in patients with CeD and their matched reference individuals*.
**Table S15**: *Sensitivity analyses of incident AP in patients with CeD and their matched reference individuals, by different exclusions and follow‐up approaches*.

## Data Availability

The data set cannot be shared directly under current legislation for data protection and must be requested directly from the respective registry holders, Statistics Sweden (information@scb.se) and the Swedish National Board of Health and Welfare (registerservice@socialstyrelsen.se), after approval by the Swedish Ethical Review Authority.
