# Supplementary material for: Long‐term risk of acute pancreatitis in patients with celiac disease: A nationwide population‐based cohort study
Source: J Intern Med. 2026 Feb 13;299(4):515–28. doi: 10.1111/joim.70074 (PMC12950633; doi:10.1111/joim.70074)
Supplement: Supplementary file 1 — Fig. S1: Graphic definition of investigated outcomes. Fig. S2: Flow chart for study population selection. Fig. S3: Alternative time intervals to define certain secondary outcomes. Table S1: Multi‐centre or population‐based evidence on AP risk in CeD. Table S2: SNOMED codes defining CeD. Table S3: Definitions of exclusion criteria. Table S4a: Definitions of outcomes and comorbidities. Table S4b: Medications for refractory CeD that may be associated with AP. Table S5: Cumulative incidence difference (95%CI) of incident AP during follow‐up in individuals with CeD, compared with their matched reference individuals. Table S6: Subgroup analyses of any incident AP in patients with CeD and their matched reference individuals. Table S7: Subgroup analyses of incident AP by different aetiologies and severe AP in patients with CeD and their matched reference individuals. Table S8: Characteristics of patients with CeD and their matched reference individuals who were at risk for recurrent AP, n (%). Table S9: Sensitivity analyses of incident AP in patients with CeD and their matched reference individuals, by alternative time intervals to define certain secondary outcomes. Table S10: Characteristics of CeD patients who had a follow‐up biopsy between 6 months and 5 years after diagnosis, n (%). Table S11: Incident AP in patients with CeD who had a follow‐up biopsy between 6 months and 5 years after diagnosis. Table S12: Characteristics of patients with CeD and their full siblings, n (%). Table S13: Sensitivity analysis of incident AP in patients with CeD and their full siblings. Table S14: Sensitivity analysis of prior AP in patients with CeD and their matched reference individuals. Table S15: Sensitivity analyses of incident AP in patients with CeD and their matched reference individuals, by different exclusions and follow‐up approaches. [file JOIM-299-515-s001.doc]

**Supplementary material**

**Long-term risk of acute pancreatitis in patients with celiac disease: a nationwide population-based cohort study**

**Yao et al.**

**Table of Contents**

[Table S1 Multi-centre or population-based evidence on AP risk in CeD 2](#__RefHeading___Toc20734)

[Table S2 SNOMED codes defining CeD ^a^ 5](#__RefHeading___Toc4040)

[Table S3 Definitions of exclusion criteria 6](#__RefHeading___Toc17037)

[Figure S1 Graphic definition of investigated outcomes 7](#__RefHeading___Toc23171)

[Table S4a Definitions of outcomes and comorbidities 8](#__RefHeading___Toc18626)

[Table 4b Medications for refractory CeD that may be associated with AP 10](#__RefHeading___Toc18130)

[Figure S2 Flow chart for study population selection 11](#__RefHeading___Toc28842)

[Table S5 Cumulative incidence difference (95%CI) of incident AP during follow-up in individuals with CeD, compared with their matched reference individuals 12](#__RefHeading___Toc19384)

[Table S6 Subgroup analyses of any incident AP in patients with CeD and their matched reference individuals 13](#__RefHeading___Toc8898)

[Table S7 Subgroup analyses of incident AP by different etiologies and severe AP in patients with CeD and their matched reference individuals 14](#__RefHeading___Toc20276)

[Table S8 Characteristics of patients with CeD and their matched reference individuals who were at risk for recurrent AP, n (%) 17](#__RefHeading___Toc13978)

[Figure S3 Alternative time intervals to define certain secondary outcomes 19](#__RefHeading___Toc30183)

[Table S9 Sensitivity analyses of incident AP in patients with CeD and their matched reference individuals, by alternative time intervals to define certain secondary outcomes 20](#__RefHeading___Toc25717)

[Table S10 Characteristics of CeD patients who had a follow-up biopsy between six months and five years after diagnosis, n (%) 21](#__RefHeading___Toc11420)

[Table S11 Incident AP in patients with CeD who had a follow-up biopsy between six months and five years after diagnosis 22](#__RefHeading___Toc3362)

[Table S12 Characteristics of patients with CeD and their full siblings, n (%) 23](#__RefHeading___Toc9044)

[Table S13 Sensitivity analysis of incident AP in patients with CeD and their full siblings 24](#__RefHeading___Toc20830)

[Table S14 Sensitivity analysis of prior AP in patients with CeD and their matched reference individuals 25](#__RefHeading___Toc28048)

[Table S15 Sensitivity analyses of incident AP in patients with CeD and their matched reference individuals, by different exclusions and follow-up approaches 26](#__RefHeading___Toc32124)

**Table S1 Multi-centre or population-based evidence on AP risk in CeD**

| **Current study** | | | | | | | | |
| --- | --- | --- | --- | --- | --- | --- | --- | --- |
| **First author, publication year,  place,  data source, study period, follow-up time** | **Sample size** | **Age at CeD diagnosis, years** | **Females, %** | **Number of outcomes in CeD** | **Incidence rate of outcome, per 100,000 person-years** | **First year of follow-up included?** | **Covariates** | **Main findings** |
| Yao, conducted in 2025, Sweden, Nationwide in- and outpatient register plus a nationwide histopathology cohort,  cases identified between 1969 and 2023, followed through August 2024,  Median follow-up: 15.5 years (IQR: 8.5-23.0) | Biopsy-confirmed CeD: 57,221 | Median: 28.4 years,  all ages included | 62.3 | Any AP: 549  Non-gallstone-related AP: 255  Gallstone-related AP: 260  Alcohol-related AP: 47  Severe AP: 107 | Any AP: 58.7  Non-gallstone-related AP: 27.3  Gallstone-related AP: 27.8  Alcohol-related AP: 5.0  Severe AP: 11.4 | Included, excluded in sensitivity analysis | (Model 1)  1. birth year 2. sex 3. county of residence 4. calendar year  +  (Model 2)  5. country of birth 6. education level 7. number of healthcare visits 8. history of autoimmune diseases before the index date | aHR for primary outcome in patients with CeD, based on Model 2 (same as below): Any AP: 1.42 (95%CI: 1.28 to 1.58)  aHRs for secondary outcomes:  Non-gallstone-related AP: 1.49 (1.27 to 1.74)  Gallstone related AP: 1.34 (1.16 to 1.55)  Alcohol-related AP: 1.20 (0.85 to 1.70)  Severe AP: 1.60 (1.25 to 2.04)  Recurrent AP: 0.85 (0.67 to 1.08) |
| **Individual studies** | | | | | | | | |
| **First author, publication year,  place,  data source, study period, follow-up time** | **Sample size** | **Age at CeD diagnosis, years** | **Females, %** | **Number of outcomes in CeD** | **Incidence rate of outcome, per 100,000 person-years** | **First year of follow-up included?** | **Covariates** | **Main findings** |
| Krishnan [1], 2025, the US, Multicenter in- and outpatient database (TriNetx), cases identified between March 2005 and April 2022, results reported at 1, 3, 5, and 7 years after CeD diagnosis | 137,685 (for any AP, after 1:1 propensity matching | <18 years:  13.5%;  18-<40: 30.5%  ≥65 years: 24.7% | 71.9 for all included individuals | any AP: 3345 at 7-year follow-up | Not reported | Included | Covariates for propensity score: 1. demographics 2. smoking 3. alcohl-related disease  4. body mass index 5. comorbid conditions  6. ERCP and cholecystectomy  7. familial hypercholesterolemia  8. Serum laboratory test results | Individuals with CeD had a higher relative risk of AP at seven years after CeD diagnosis, HR=2.05 (95% CI: 1.93 to 2.17);  For idiopathic AP: HR=1.49 (1.21 to 1.84);  alcoholic AP: HR=1.35 (1.03 to 1.77);  biliary AP: HR=1.37 (1.12 to 1.66). |
| [Abstract only] Krishnan [2], 2022, the US, Multicenter in- and outpatient database (TriNetx), cases identified between March 2005 and April 2022, results reported at three years after CeD diagnosis | 140,528 (for any AP, after propensity matching at three years after CeD diagnosis) | Only those >18 years included, mean 41.7 years for all individuals | 70.7 for all included individuals | any AP: 268 (0.19%) | Not reported | Included | Covariates for propensity score: 1. demographics 2. smoking 3. alcohl-related disease 4. diabetes 5. hyperlipidemia 6. comorbid conditions | Individuals with CeD had a higher relative risk of AP at three years after CeD diagnosis, RR=1.30 (95% CI: 1.08 to 1.56) |
| Alkhayyat [3], 2021, the US, Multicenter in- and outpatient database (IBM Explorys Inc.,),  cases identified between 1999 and May 2020,  follow-up time not reported | 133,400 | <18 years:  5.6%  >65 years: 25.1% | 75.3 | Event number not reported. Prevalence of first AP episode after 30 days of CeD diagnosis was 1.06% | N/A | Included, but the first 30 days were excluded | 1. age 2. gender 3. race 4. alcohol abuse 5. smoking 6. idiopathic pancreatitis 7. alcohol-induced pancreatitis 8. gallstone-induced pancreatitis | Patients with CeD had higher odds of developing AP after at least 30 days of CeD diagnosis (aOR=2.66, 95%CI: 2.55 to 2.77), of which:  For idiopathic AP: aOR=1.54 (1.34 to 1.77);  alcoholic AP: aOR=1.02 (0.90 to 1.16);  gallstone AP: aOR=1.25 (0.75 to 2.09).  In addition, patients with CeD also had a higher risk of developing complications  both within seven days after an AP episode (including acute respiratory distress syndrome [prevalence, CeD vs. non-CeD: 0.33% vs. 0.17%], abdominal compartment syndrome [0.17% vs. 0.07%], ileus [0.66% vs. 0.29%], and hypocalcemia [2.97% vs. 2.05%]) (all P<0.05).  There was also a higher risk of complications within thirty days after an AP episode (including ascites [4.95% vs. 3.49%], gastrointestinal bleeding [14.52% vs. 5.17%], hospital admission [44.21% vs. 41.73%], strokes [5.28% vs. 0.29%], pneumonia [9.57% vs. 5.18%], and pulmonary embolism [5.28% vs. 1.04%]) (all P<0.05). |
| Osagiede [4], 2020, the US, nationwide inpatient database (the Nationwide Inpatient Sample),  Inpatients records between 2007 and 2016,  Follow-up not reported. | 337,201 | Mean: 53.3 years for overall patients with CeD; 50.9 years for patients with CeD+AP | 71 (for overall patients with CeD); 68 (patients with CeD+AP) | 7,372 (2.2%) in hospitalized patients with CeD; prevalence by calendar years ranged from 1.01% in 2007 to 2.9% in 2016 | N/A | N/A | Covariates for propensity score: 1. age 2. sex 3. ethnicity 4. median neighborhood income 5. Charlson comorbidity index 6. hospital region 7. location 8. number of beds + also adjusted for 9. insurance carrier 10. hospital teaching status 11. weekend admission | There was higher inpatient prevalence of any AP in CeD vs. non-CeD: 2.2% vs. 1.2% (P < 0.01).  Any AP occurred more frequently during hospitalization of patients with CeD (aOR=1.92, 95%CI: 1.82 to 2.05), of which,  for alcoholic AP, aOR=0.45 (0.06 to 3.22);  biliary AP: aOR=0.51 (0.43 to 0.59);  hypertriglyceridemia AP: aOR=0.78 (0.48 to 1.12);  hypercalcemia AP: aOR=0.85 (0.38 to 1.91);  post-ERCP: aOR=0.46 (0.38 to 0.55);  others: aOR=2.44 (2.16 to 2.76)  However, there were lower odds of morbidity (including admission to the intensive care unit, shock, and multiorgan failure) and mortality in AP patients with CeD than those without. |
| Sadr-Azodi [5], 2012, Sweden, nationwide in- and outpatient register, plus a nationwide histopathology cohort,  case identified between 1969 and 2008 and followed through 2009.  Median follow-up for *any* pancreatitis ^a^: 10 years (range: 0-41 years) | 28,908 | Median: 30 years (range: 0-95 years) | 62.0 | Gallstone-related AP: 30  Non-gallstone-related AP:133 | Gallstone-related AP: 9;  Non-gallstone-related AP: 41 | Included, excluded in sensitivity analysis | Matching variables  1. age 2. sex 3. calendar year 4. residence county | Patients with CeD had a higher hazard of developing gallstone-related AP (aHR=1.59, 95%CI: 1.06 to 2.40) and non-gallstone-related AP (aHR=1.86, 95%CI: 1.52 to 2.26]). The relative risk for severe AP (defined as hospital stay ≥14 days or in-hospital mortality) ^a^ was also increased. The aHR for gallstone-related severe AP was 3.18 (1.46 to 6.90), and for non–gallstone related severe AP was 2.00 (1.16 to 3.44).  After excluding the first year of follow-up, the aHR for gallstone-related AP was 1.55 (1.02 to 2.36) and for non-gallstone-related AP was 1.74 (1.41 to 2.15). |
| Ludvigsson [6], 2007, Sweden, nationwide inpatient register,  case identified between 1964 and 2003 and followed through 2003.  Median follow-up for *any* pancreatitis ^b^: 5 years (range: 1-27 years) | 14,239 | 0-15 years: 65.8%; median: 2 years (range: 0-94 years) | 58.9 | Any AP: 67 | Not reported | Excluded | Matching variables  1. age 2. sex 3. calendar year 4. residence county | Patients hospitalized for CeD were associated had a higher hazard of AP compared with matched reference individuals (aHR: 2.5, 95%CI: 1.8 to 3.3) |

AP: acute pancreatitis; CeD: celiac disease; CI: confidence interval; ERCP: Endoscopic retrograde cholangiopancreatography; (a)HR: (adjusted) hazard ratio; IQR: interquartile range; N/A: not applicable; (a)OR: (adjusted) odds ratio; RR: relative risk

^a^ In current study, severe AP included i) hospitalization for incident AP that lasts for ≥14 days, ii) being combined with diagnostic or procedural code implying a complicated episode, **or** iii) death from any cause <90 days after being discharged for incident AP.

^b^ Any pancreatitis in the study by Sadr-Azodi et al included acute pancreatitis, chronic pancreatitis, and pancreatic enzyme supplementation[5]; while the study by Ludvigsson et al, defined any pancreatitis as acute pancreatitis and chronic pancreatitis [6].

| **Table S2 SNOMED codes defining CeD ^a^** | | |
| --- | --- | --- |
| **Condition** | **Topographic codes** | **SNOMED codes ^b^** |
| Celiac disease (villus atrophy) | Duodenum: T64 (all);  Jejunum: T65, T65000 and T651 (excluding T652-9) | Celiac diagnosis: D6218x;  Marsh 3: M58, M5800, M58000, M58001, M58005, M58006, M58007 |
| Mucosal healing | T64, T65, T65000, T651 | Marsh 0: M00100, M00110,  Marsh 1-2: M40000, M41000, M42000, M43000, M47000, M47170 |
| CeD: celiac disease; SNOMED: Systematized Nomenclature of Medicine;  ^a^ Diagnosis criterion: ≥1 SNOMED code for VA in the small intestine other than the ileum (PPV: 95% for biopsy samples between 1969 and 2008, and 99% for biopsy samples between 2009 and 2017)[7, 8].  ^b^ In SNOMED codes, diagnostic codes that were listed under morphology in pathology registers starts with “D”. For example D6218 was the diagnostic code for CeD. Meanwhile, codes that start with “M” (suggestive of inflammation but was not specific) in the table refer to all SNOMED codes starting with the respective code, which shall be accompanied by a topographic code.  The presence of persistent VA (Marsh 3) or mucosal healing (Marsh 0-2) was determined based on the follow-up biopsy result between six months and five years after the first biopsy that indicates Marsh 3 small intestinal VA. | | |

**Table S3 Definitions of exclusion criteria**

| **Exclusion** | **Data source** | **ICD-8 (1969-1986)** | **ICD-9 (1987-1996)** | **ICD-10 (1997-)** | **ICD-7 (for the Cancer register)** | **ATC** |
| --- | --- | --- | --- | --- | --- | --- |
| Non-malignant pancreatic disease or its complications | NPR, PDR | 269; 577 | 577; 579E | K85; K86; K903 | - | A09AA02 |
| Chronic pancreatitis | NPR | 577,1 | 577B | K860; K861 | - | - |
| Pancreatic cancer | NPR, the Cancer Register | 157 | 157 | C25 | 157 | - |
| Cystic fibrosis | NPR | 273,00 | 277A | E84 | - | - |
| Congenital anomalies of the pancreas or the gallbladder | NPR | 751,6; 751,7 | 751G; 751H | Q440-Q445; Q450-Q453 | - | - |
| Diagnostic or surgical procedures in the pancreas or cholecystectomy | NPR | KVÅ for procedure: 55-(procedures in the pancreas); 5350-5352; 5356; 5357; 5359 (cholecystectomy); 5388; 5394; 9014 (ERCP) | KVÅ for procedure: 55- (procedures in the pancreas); 5350-5352; 5356; 5357; 5359 (cholecystectomy); 5388; 5394; 9014 (ERCP) | KVÅ for procedure: JLA-JLD (procedures in the pancreas); JKA20-JKA21 (cholecystectomy); UJK02; UJK05; UJK12; UJK15 (ERCP) | - | - |

ATC: anatomical therapeutic chemical; CeD: celiac disease; ERCP: Endoscopic retrograde cholangiopancreatography; ICD: International Classification of Disease; KVÅ: Klassifikation av vårdåtgärder [Classification of surgical procedures]; NPR: National Patient Register; PDR: Prescribed Drug Register.

**Figure S1 Graphic definition of investigated outcomes**

**AP: acute pancreatitis; CeD: celiac disease**

**
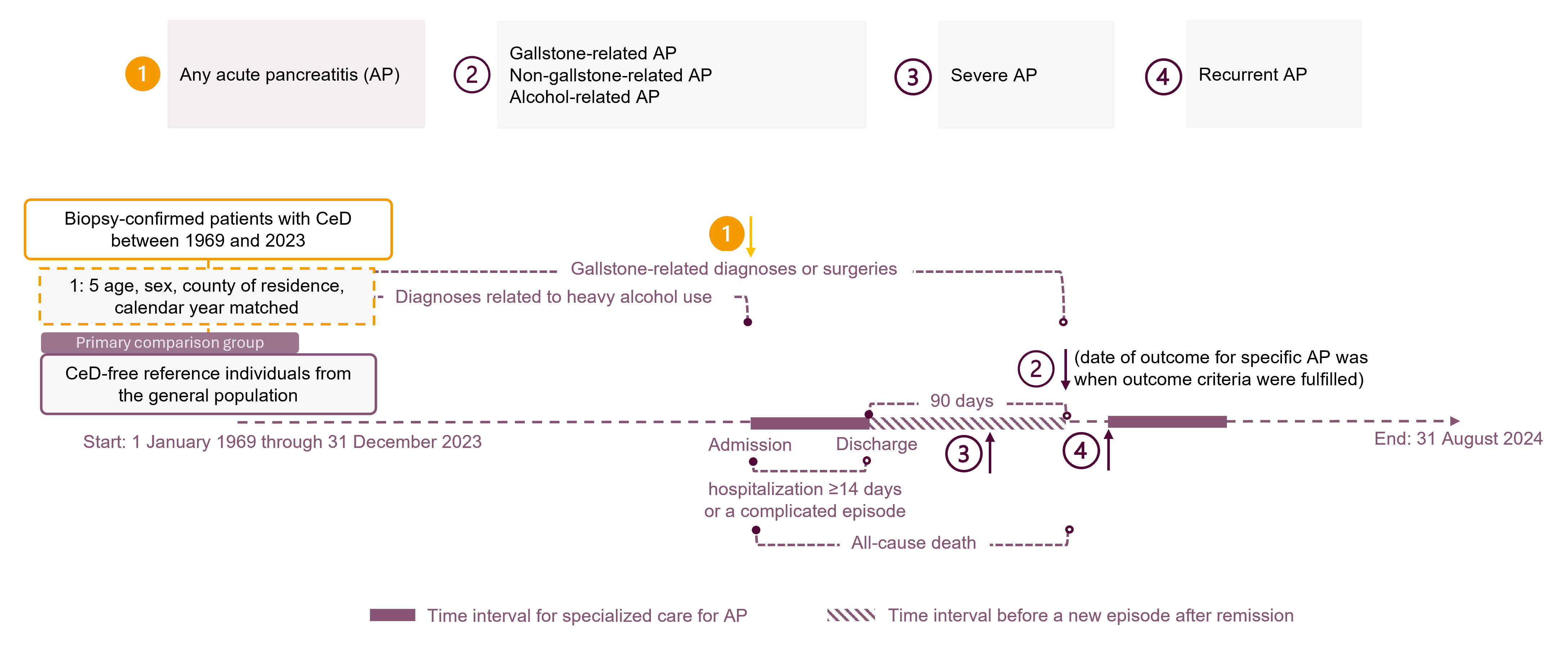
**

**Table S4a Definitions of outcomes and comorbidities**

| **Outcomes** | **Data source** | **ICD-8**  **(1969-1986)** | **ICD-9**  **(1987-1996)** | **ICD-10**  **(1997-)** | **ATC** |  | | | | |
| --- | --- | --- | --- | --- | --- | --- | --- | --- | --- | --- |
| Primary outcome | | | | | |  | | | | |
| Any AP | NPR | 577,00; 577,01; 577,02; 577,08 | 577A | K85 | - |  | | | | |
| Secondary outcomes | | | | | |  | | | | |
| Gallstone-related AP | NPR | 577,00; 577,01; 577,02; 577,08  **and**  **any** diagnostic or procedural code before incident AP or <90 days after its discharge:  ICD: 574; 575;  KVÅ: 5300; 5302; 5304; 5350-5352; 5356-5357; 5359; 5388; 5394; 9014 | 577A  **and**  **any** diagnostic or procedural code before incident AP or <90 days after its discharge:  ICD: 574; 575;  KVÅ: 5300; 5302; 5304; 5350-5352; 5356-5357; 5359; 5388; 5394; 9014 | K851;  **or**  K850; K852-K859 + **any** diagnostic or procedural code before incident AP or <90 days after its discharge:  ICD: K80, K851;  KVÅ: JKA20-JKA21; JKB00; JKB01; JKB11**;** JKE00; JKE02**;** JKE12; JKE15; JKE18; JKE25; JKE98; UJK02; UJK05; UJK12; UJK15 | - |  | | | | |
| Non-gallstone-related AP | NPR | 577,00; 577,01; 577,02; 577,08  **and**  **none** of the following diagnostic or procedural code before incident AP or <90 days after its discharge:  ICD: 574; 575;  KVÅ: 5300; 5302; 5304; 5350-5352; 5356-5357; 5359; 5388; 5394; 9014 | 577A  **and**  **none** of the following diagnostic or procedural code before incident AP or <90 days after its discharge:  ICD: 574; 575;  KVÅ: 5300; 5302; 5304; 5350-5352; 5356-5357; 5359; 5388; 5394; 9014 | K850; K852-K859;  **and**  **none** of the following diagnostic or procedural code before incident non-gallstone-related AP or <90 days after its discharge:  ICD: K80; K851;  KVÅ: JKA20-JKA21; JKB00; JKB01; JKB11**;** JKE00; JKE02**;** JKE12; JKE15; JKE18; JKE25; JKE98; UJK02; UJK05; UJK12; UJK15 | - |  | | | | |
| Alcohol-related AP | NPR, PDR | Non-gallstone-related AP **and history of heaving alcohol consumption:** 261,00; 262,00; 280,00; 281,00; 291; 291,1; 303; 307,00; 307,10; 307,99; 322; 571,00; 571,01; 581,10; 583,10; 979; 980,00; 980,01; 980,98; 980,99 | Non-gallstone-related AP **and history of heaving alcohol consumption:** 291; 294A; 303; 305A; 357F; 425F; 535D; 571A-D; 760W; 790D; 977D; 980A; 980X; V97B | K852 **or** K850; K858; K859 (non-gallstone-related AP) + **any code for a history of heaving alcohol consumption:** ICD: E244; F10; G312; G621; G721; I426; K292; K70; O354; R780; T510; T518; T519; X65; Y15; Y573; Y90; Y91; Z502; Z714; Z721 ATC: N07BB01; N07BB03; N07BB04 | - |  | | | | |
| Severe acute pancreatitis | NPR | Hospitalization for incident AP that lasts for ≥14 days **or** being combined with diagnostic or procedural code implying a complicated episode, **or** death from any cause <90 days after being discharged for incident AP (see ICD codes above) | | | |  | | | | |
| Acute respiratory failure |  | ICD: 519; 783,1; Y20;  KVÅ: 0020; 2250; 2251; 2260; 9122; 9126-30; 9201 | ICD: 518F; 799B; V44A; V55A;  KVÅ: 0020; 2250; 2251; 2260; 9122; 9126-30; 9201 | ICD: J960; J969; Z430; Z930  KVÅ: DG001; DG002; DG004; DG007; DG010; DG016; DG019-27; GBB00; GBB03; GBB13; GBB16; GBB18 | - |  | | | | |
| Acute renal failure |  | ICD: 593,1; 593,2  KVÅ: 9210; 9211; 9219; 9223 | ICD: 584; 586X; V56A;  KVÅ: 9210; 9211; 9219; 9223 | ICD: N17; Z490; Z491  KVÅ: DK001; DK005; DP002; DR013-15; DR017; DR018; DR020; DR055; DR056; DR061; SP381; SP382 | - |  | | | | |
| Abdominal compartment syndrome |  | KVÅ: 0060; 0061; 0062; 3411; 3414; 3500;4030; 4031; 5500 | KVÅ: 0060; 0061; 0062; 3411; 3414; 3500;4030; 4031; 5500 | ICD: R198A;  KVÅ: GAA10; GAA96; GAA97; TGA30; TGA35; DG003; DG014; JAH30; JAH33; JLA00; JAK00; JAK01; JAK03; JAK04; JLB38; JLB96; JLB98; JLD10; JLD12; JLD20; JLD22; TJA40; TJL10; ZXL00; ZXL10 | - |  | | | | |
| **Comorbidities** | **Data source** | **ICD-8**  **(1969-1986)** | **ICD-9**  **(1987-1996)** | **ICD-10**  **(1997-)** | **ATC** |  | | | | |
| Autoimmune diseases | | | | | |  |  |  |  |  |
| Autoimmune liver diseases | NPR | 571,9; 573,0; 575,05 | 571E; 573D; 571G; 576B | K743; K745; K754; K830A | - |  | | | | |
| Systemic lupus erythematosus/cutaneous lupus erythematosus | NPR | 734,1 | 710A | M32; L931 | - |  | | | | |
| Vitiligo | NPR | 709,05 | - | L80 | - |  | | | | |
| Psoriasis | NPR | 696,0; 696,1 | 696A; 696B | L40 | - |  | | | | |
| Alopecia areata | NPR | 704,00 | - | L63 | - |  | | | | |
| Systemic sclerosis | NPR | 734,00-734,19 | 710B | M34 | - |  | | | | |
| Dermatomyositis or juvenile dermatomyositis | NPR | 716,00 | 710D | M33 except M332 | - |  | | | | |
| Polymyositis | NPR | 716,10 | 710E | M332 | - |  | | | | |
| Type 1 diabetes | NPR | 250 (only if the first diagnosis ≤30 years ^a^) | 250 (only if the first diagnosis ≤30 years ^a^) | E10 | - |  | | | | |
| Autoimmune thyroid disease | NPR | 242,00; 242,09; 244; 245,02; 245,03 | 242A; 242X; 244X; 245C; 245W | E035; E039; E050; E055; E059; E063; E065 | - |  | | | | |
| Addison’s disease | NPR | 255,10 | 255E | E271; E272 | - |  | | | | |
| Myasthenia gravis | NPR | 733,00 | 358A | G700 | - |  | | | | |
| Inflammatory bowel disease | NPR | 563; 563,0; 563,00; 563,1; 563,10; 563,9; 563,98; 563,99; 569,02; 569,04 | 555; 556 | K50; K51; K523 | - |  | | | | |
| Sjögren syndrome | NPR | 734,90 | 710C | M350 | - |  | | | | |
| Lambert-Eaton syndrome | NPR | - | - | G731 | - |  | | | | |
| Rheumatoid arthritis | NPR | 712,10; 712,20; 712,38; 712,39 | 714A; 714B; 714C; 714W; 719D | M05; M060; M062; M063; M068; M069; M123 | - |  | | | | |
| Juvenile arthritis | NPR | 712,0 | 714D | M08 | - |  | | | | |
| Multiple sclerosis | NPR | 340,99 | 340 | G35 | - |  | | | | |
| Ankylosing spondylitis | NPR | 712,40 | 720A | M45 | - |  | | | | |
| Spondyloarthritis | NPR | 713,13; 726,99 | 720A; 696A; 713B; 099D; 711A; 720B; 720C; 720W | M45; M081; L405; M070-M076; M0912; M020-M023; M028-M029; M460; M461; M468; M469 | - |  | | | | |
| Sarcoidosis | NPR | 135 | 135 | D86; G532; M633 | - |  | | | | |
| Metabolic-related diseases |  |  |  |  |  |  | | | | |
| Hypertension | NPR, PDR | 400-404 | 401-405 | I10; I119; I12-I15; | C02; C03AA-AB; C03BA; C03CA; C03DA; C03EA; C08CA; C08DA; C08DB; C09A; C09BA; C09BB; C09CA; C09DA; C09DB01 |  | | | | |
| Diabetes | NPR, PDR | 250 | 250 | E10-E14; O24 | A10 |  | | | | |
| Obesity | NPR | 277 | 278A; 278B | E65-E66 | - |  | | | | |
| Dyslipidemia | NPR, PDR | 279 | 272 | E78 | C10AA; C10AB; C10AC; C10AD; C10AX01-14 |  | | | | |
| Chronic obstructive pulmonary disease (as a proxy for heavy smoking only if diagnosed ≥40 years) ^b^ | NPR | 491; 492 | 491; 492; 496 | J41-J44 | - |  | | | | |
| Heavy alcohol use [9] | NPR, PDR | 261,00; 262,00; 280,00; 281,00; 291; 291,1; 303; 307,00; 307,10; 307,99; 322; 571,00; 571,01; 581,10; 583,10; 979; 980,00; 980,01; 980,98; 980,99 | 291; 294A; 303; 305A; 357F; 425F; 535D; 571A-D; 760W; 790D; 977D; 980A; 980X; V97B | E244; F10; G312; G621; G721; I426; K292; K70; O354; R780; T510; T518; T519; X65; Y15; Y573; Y90; Y91; Z502; Z714; Z721 | N07BB01; N07BB03; N07BB04 (alcohol abuse treatment) |  | | | | |

AP: acute pancreatitis; ATC: anatomical therapeutic chemicals; CeD: celiac disease; ICD: International Classification of Disease; KVÅ: Klassifikation av vårdåtgärder [Classification of surgical procedures]; NPR: the National Patient Register; PDR: the Prescribed Drug Register.

^a^ The algorithm for identifying the diagnosis of type 1 diabetes via ICD-8 and ICD-9 was proved to have a high positive predictive value (>95%)[10].

^b^ As in our earlier research [11], for chronic obstructive pulmonary disease to be a proxy for heavy smoking, we restricted patients diagnosed after 40 years of age to avoid misclassification due to genetic susceptibility.

| **Table 4b Medications for refractory CeD that may be associated with AP[12-14]** | |
| --- | --- |
| **Medications** | **ATC code** |
| Steroids | A07EA06 (budesonide); H02AB07; A07EA03 (prednisone) |
| Mesalamine | A07EC02 |
| Immunosuppressants | L04AX01 (azathioprine); L01BB02 (mercaptopurine) |
| AP: acute pancreatitis; ATC: Anatomical Therapeutic Chemical; CeD: celiac disease. | |

**Figure S2 Flow chart for study population selection**

**CeD: celiac disease; ESPRESSO: the Epidemiology Strengthened by histoPathology Reports in Sweden.**

**
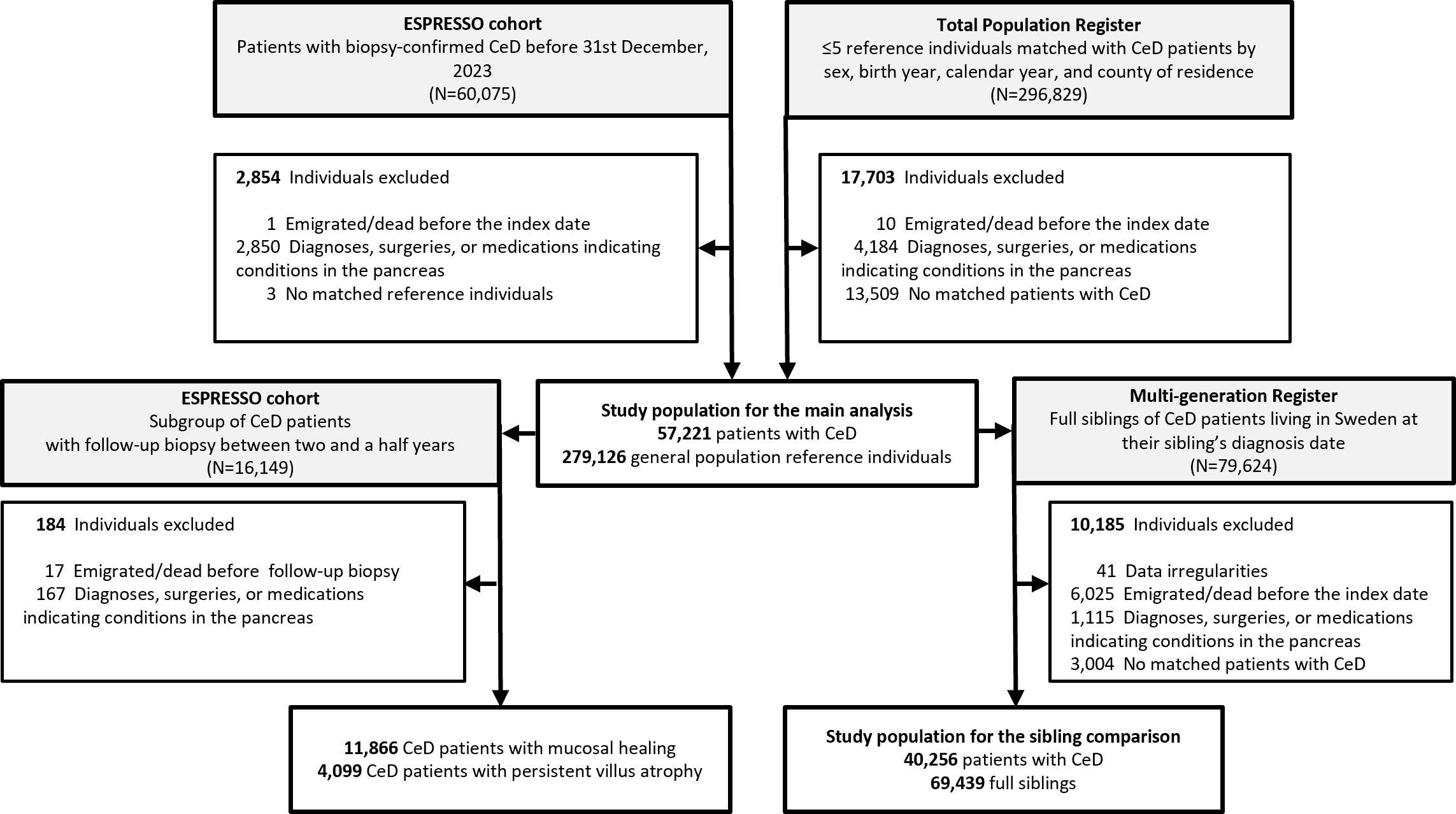
**

**Table S5 Cumulative incidence difference (95%CI) of incident AP during follow-up in individuals with CeD, compared with their matched reference individuals**

| **Outcomes** | **Difference in standardized cumulative incidence in percentages (95%CI), years since the index date** ^a^ | | | |
| --- | --- | --- | --- | --- |
|  | **1 year** | **5 years** | **10 years** | **25 years** |
| Primary outcome | | | | |
| Any AP | 0.03 (0.01 to 0.04) | 0.09 (0.05 to 0.13) | 0.18 (0.12 to 0.24) | 0.54 (0.36 to 0.72) |
| Secondary outcomes | | | | |
| Gallstone-related AP | 0.000 (-0.007 to 0.007) | 0.01 (-0.01 to 0.03) | 0.06 (0.01 to 0.10) | 0.31 (0.15 to 0.46) |
| Non-gallstone-related AP | 0.02 (0.01 to 0.03) | 0.07 (0.04 to 0.10) | 0.11 (0.07 to 0.16) | 0.21 (0.09 to 0.32) |
| Alcohol-related AP | 0.003 (0.000 to 0.007) | 0.008 (-0.004 to 0.02) | 0.01 (-0.008 to 0.03) | 0.008 (-0.06 to 0.07) |
| Severe AP | 0.007 (0.000 to 0.014) | 0.03 (0.01 to 0.05) | 0.05 (0.02 to 0.08) | 0.13 (0.04 to 0.22) |
| AP: acute pancreatitis; CeD: celiac disease; CI: confidence interval | | | | |
| ^a^ Model 2: conditioned on the matching variables (birth year, sex, county of residence, and calendar year of index date) and further adjusted for country of birth, educational attainment, number of healthcare visits between two years and six months before the index date, and the history of autoimmune diseases. | | | | |

| **Table S6 Subgroup analyses of any incident AP in patients with CeD and their matched reference individuals** | | | | | |  |
| --- | --- | --- | --- | --- | --- | --- |
| **Subgroup** | | **No. of events**  **(IR, per 100,000 person-years)** | | **IR difference (95%CI), per 100,000 person-years** | **HR (95%CI)** | |
|  |  | **Patients** | **References** |  | **Model 1 ^a^** | **Model 2 ^b^** |
| Age at index date | |  |  |  |  |  |
| <18 | | 71 (17.2) | 258 (12.7) | 4.5 (0.2 to 8.8) | 1.34 (1.03 to 1.74) | 1.22 (0.92 to 1.62) |
| 18-<40 | | 123 (56.3) | 364 (34.0) | 22.2 (11.7 to 32.8) | 1.64 (1.33 to 2.02) | 1.46 (1.17 to 1.83) |
| 40-<60 | | 182 (93.5) | 578 (60.8) | 32.7 (18.3 to 47.2) | 1.58 (1.33 to 1.88) | 1.46 (1.22 to 1.75) |
| ≥60 | | 173 (157.6) | 532 (99.9) | 57.8 (32.8 to 82.7) | 1.51 (1.26 to 1.82) | 1.46 (1.21 to 1.76) |
| Sex | |  |  |  |  |  |
| Female | | 307 (51.8) | 1004 (34.8) | 17.0 (10.8 to 23.2) | 1.47 (1.29 to 1.68) | 1.35 (1.18 to 1.55) |
| Male | | 242 (70.7) | 728 (43.0) | 27.7 (18.2 to 37.1) | 1.63 (1.40 to 1.90) | 1.52 (1.30 to 1.79) |
| Born in Nordic countries ^c^ |  | | |  |  |  |
| Yes | | 523 (58.2) | 1641 (37.3) | 20.9 (15.6 to 26.2) | 1.55 (1.40 to 1.71) | 1.46 (1.31 to 1.62) |
| No | | 26 (72.6) | 91 (50.0) | 22.6 (-7.2 to 52.3) | 1.35 (0.86 to 2.11) | 1.18 (0.57 to 2.45) |
| Calendar period at index date |  | | |  |  |  |
| 1969-1989 | | 57 (57.4) | 216 (44.1) | 13.3 (-2.7 to 29.4) | 1.30 (0.96 to 1.77) | 1.12 (0.80 to 1.56) |
| 1990-2001 | | 241 (61.9) | 740 (38.7) | 23.3 (15.0 to 31.6) | 1.61 (1.38 to 1.88) | 1.48 (1.27 to 1.74) |
| 2002-2011 | | 186 (58.0) | 583 (37.3) | 20.6 (11.8 to 29.5) | 1.53 (1.29 to 1.81) | 1.40 (1.17 to 1.67) |
| 2012-2023 | | 65 (51.8) | 193 (31.6) | 20.3 (6.9 to 33.6) | 1.56 (1.17 to 2.07) | 1.48 (1.10 to 1.99) |
| Educational attainment, years |  | | |  |  |  |
| 0-9 | | 159 (139.1) | 387 (68.3) | 70.8 (48.1 to 93.4) | 2.15 (1.77 to 2.62) | 1.88 (1.47 to 2.41) |
| 10-12 | | 199 (51.0) | 653 (34.5) | 16.4 (8.9 to 24.0) | 1.44 (1.22 to 1.70) | 1.14 (0.94 to 1.38) |
| ≥13 | | 138 (36.9) | 487 (26.7) | 10.2 (3.6 to 16.8) | 1.30 (1.07 to 1.58) | 1.49 (1.17 to 1.91) |
| Missing | | 53 (93.8) | 205 (68.3) | 25.5 (-1.4 to 52.5) | 1.40 (1.01 to 1.94) | 1.15 (0.80 to 1.65) |
| History of metabolic-related diseases ^d,e^ | | | |  |  |  |
| Yes | | 79 (95.5) | 267 (62.4) | 33.1 (10.8 to 55.5) | 1.52 (1.17 to 1.98) | 1.36 (1.01 to 1.82) |
| No | | 470 (55.2) | 1465 (35.3) | 19.9 (14.6 to 25.2) | 1.54 (1.38 to 1.71) | 1.45 (1.30 to 1.62) |
| History of autoimmune diseases ^d^ | | | |  |  |  |
| Yes | | 100 (113.1) | 219 (48.4) | 64.7 (41.6 to 87.8) | 2.58 (2.01 to 3.31) | 0.99 (0.53 to 1.87) |
| No | | 449 (53.0) | 1513 (36.7) | 16.4 (11.1 to 21.6) | 1.41 (1.26 to 1.57) | 1.39 (1.24 to 1.55) |
| History of heavy alcohol consumption ^d^ | |  |  |  |  |  |
| Yes | | 22 (215.2) | 35 (56.9) | 158.3 (66.4 to 250.1) | 4.32 (2.39 to 7.83) | 3.79 (1.75 to 8.23) |
| No | | 527 (57.0) | 1697 (37.6) | 19.4 (14.2 to 24.6) | 1.50 (1.35 to 1.66) | 1.39 (1.25 to 1.54) |
| History of COPD≥40 years ^d,f^ | |  |  |  |  |  |
| Yes | | 6 (228.1) | 18 (99.9) | 128.2 (-60.0 to 316.4) | 1.72 (0.61 to 4.85) | 1.33 (0.29 to 6.04) |
| No | | 543 (58.3) | 1714 (37.6) | 20.7 (15.5 to 25.9) | 1.54 (1.39 to 1.70) | 1.42 (1.28 to 1.58) |
| AP: acute pancreatitis; CeD: celiac disease; CI: confidence interval; COPD: chronic obstructive pulmonary disease; HR: hazard ratio; IR: incidence rate  ^a^ Model 1: conditioned on the matching variables (birth year, sex, county of residence, and calendar year).  ^b^ Model 2: model 1 and further adjusted for country of birth, educational attainment, number of healthcare visits between two years and six months before the index date, and history of autoimmune diseases.  ^c^ Nordic countries: Sweden, Denmark, Finland, Norway, and Iceland  ^d^ Codes for disease histories are listed in Supplementary Table S4  ^e^ Metabolic-related diseases: hypertension, diabetes, obesity, and dyslipidemia  ^f^ Proxy for heavy smoking [11] | | | | | | |

**Table S7 Subgroup analyses of incident AP by different etiologies and severe AP in patients with CeD and their matched reference individuals**

|  | **No. of events**  **(IR, per 100,000 person-years)** | | **IR difference (95%CI), per 100,000 person-years** | **HR (95%CI)** | | |
| --- | --- | --- | --- | --- | --- | --- |
| **Subgroup** | **Patients** | **References** |  | **Model 1 ^a^** | | **Model 2 ^b^** |
|  | **Gallstone-related AP** | | | | | |
| Age at index date |  |  |  |  | |  |
| <18 | 21 (5.1) | 134 (6.6) | -1.5 (-4.0 to 0.9) | 0.77 (0.48 to 1.22) | | 0.76 (0.47 to 1.23) |
| 18-<40 | 61 (27.8) | 197 (18.4) | 9.5 (2.0 to 16.9) | 1.50 (1.12 to 2.01) | | 1.41 (1.04 to 1.92) |
| 40-<60 | 92 (47.1) | 288 (30.2) | 16.9 (6.7 to 27.1) | 1.60 (1.25 to 2.04) | | 1.51 (1.18 to 1.95) |
| ≥60 | 86 (78.0) | 279 (52.2) | 25.7 (8.2 to 43.3) | 1.43 (1.10 to 1.86) | | 1.43 (1.09 to 1.87) |
| Sex |  |  |  |  | |  |
| Female | 169 (28.5) | 598 (20.7) | 7.8 (3.2 to 12.4) | 1.36 (1.14 to 1.62) | | 1.29 (1.07 to 1.54) |
| Male | 91 (26.5) | 300 (17.7) | 8.8 (3.0 to 14.6) | 1.49 (1.16 to 1.91) | | 1.46 (1.13 to 1.90) |
| Born in Nordic countries ^c^ | | |  |  | |  |
| Yes | 248 (27.5) | 847 (19.2) | 8.3 (4.6 to 11.9) | 1.42 (1.22 to 1.64) | | 1.39 (1.19 to 1.62) |
| No | 12 (33.4) | 51 (28.0) | 5.5 (-15.0 to 25.9) | 1.08 (0.57 to 2.05) | | 0.77 (0.28 to 2.14) |
| Calendar period at index date | | |  |  | |  |
| 1969-1989 | 26 (26.1) | 122 (24.8) | 1.3 (-9.7 to 12.2) | 1.03 (0.66 to 1.61) | | 0.97 (0.60 to 1.55) |
| 1990-2001 ^d^ | 117 (30.0) | 369 (19.3) | 10.7 (5.0 to 16.5) | 1.56 (1.25 to 1.94) | | 1.52 (1.22 to 1.90) |
| 2002-2011 | 85 (26.4) | 301 (19.2) | 7.2 (1.2 to 13.2) | 1.40 (1.09 to 1.79) | | 1.31 (1.01 to 1.70) |
| 2012-2023 | 32 (25.5) | 106 (17.3) | 8.2 (-1.3 to 17.6) | 1.34 (0.90 to 2.00) | | 1.35 (0.89 to 2.05) |
| Educational attainment, years | | |  |  | |  |
| 0-9 | 75 (65.3) | 198 (34.9) | 30.4 (14.9 to 46.0) | 2.07 (1.56 to 2.75) | | 1.82 (1.27 to 2.59) |
| 10-12 | 97 (24.8) | 332 (17.5) | 7.3 (2.0 to 12.5) | 1.34 (1.06 to 1.69) | | 1.09 (0.83 to 1.43) |
| ≥13 | 63 (16.8) | 255 (14.0) | 2.9 (-1.7 to 7.4) | 1.15 (0.87 to 1.53) | | 1.37 (0.96 to 1.95) |
| Missing | 25 (44.1) | 113 (37.5) | 6.6 (-12.1 to 25.2) | 1.14 (0.72 to 1.81) | | 1.08 (0.66 to 1.77) |
| History of metabolic-related diseases ^e,f^ | | |  |  | |  |
| Yes | 35 (42.2) | 135 (31.5) | 10.7 (-4.2 to 25.7) | 1.36 (0.92 to 2.02) | | 1.30 (0.83 to 2.02) |
| No | 225 (26.3) | 763 (18.4) | 8.0 (4.3 to 11.7) | 1.40 (1.20 to 1.64) | | 1.36 (1.16 to 1.59) |
| History of autoimmune diseases ^e^ | | |  |  | |  |
| Yes | 37 (41.7) | 118 (26.1) | 15.6 (1.4 to 29.8) | 1.74 (1.19 to 2.56) | | 0.85 (0.35 to 2.09) |
| No | 223 (26.3) | 780 (18.9) | 7.4 (3.7 to 11.1) | 1.35 (1.16 to 1.58) | | 1.32 (1.13 to 1.55) |
| History of heavy alcohol consumption ^e^ |  |  |  |  | |  |
| Yes | 7 (67.8) | 16 (26.0) | 41.9 (-10.0 to 93.7) | 4.13 (1.47 to 11.60) | | 4.03 (0.87 to 18.62) |
| No | 253 (27.3) | 882 (19.5) | 7.8 (4.2 to 11.4) | 1.37 (1.19 to 1.59) | | 1.32 (1.14 to 1.54) |
| History of COPD≥40 years ^e,g^ |  |  |  |  | |  |
| Yes | 3 (113.8) | 9 (49.8) | 64.0 (-68.8 to 196.9) | 2.94 (0.64 to 13.41) | | 2.60 (0.07 to 92.33) |
| No | 257 (27.5) | 889 (19.5) | 8.0 (4.4 to 11.6) | 1.39 (1.20 to 1.61) | | 1.33 (1.15 to 1.55) |
|  | **Non-gallstone-related AP** | | | | | |
| Age at index date |  |  |  |  | |  |
| <18 | 47 (11.4) | 123 (6.1) | 5.3 (1.9 to 8.8) | 1.85 (1.32 to 2.60) | | 1.64 (1.14 to 2.37) |
| 18-<40 | 53 (24.2) | 157 (14.7) | 9.6 (2.7 to 16.5) | 1.63 (1.19 to 2.23) | | 1.26 (0.89 to 1.81) |
| 40-<60 | 82 (42.1) | 257 (27.0) | 15.1 (5.4 to 24.8) | 1.68 (1.30 to 2.18) | | 1.51 (1.15 to 1.99) |
| ≥60 | 73 (66.5) | 209 (39.2) | 27.3 (11.1 to 43.4) | 1.69 (1.27 to 2.24) | | 1.59 (1.18 to 2.14) |
| Sex |  |  |  |  | |  |
| Female | 119 (20.1) | 367 (12.7) | 7.4 (3.5 to 11.2) | 1.63 (1.32 to 2.02) | | 1.38 (1.10 to 1.73) |
| Male | 136 (39.7) | 379 (22.4) | 17.3 (10.3 to 24.4) | 1.77 (1.45 to 2.17) | | 1.58 (1.27 to 1.95) |
| Born in Nordic countries ^c^ | | |  |  | |  |
| Yes | 243 (27.0) | 709 (16.1) | 10.9 (7.3 to 14.5) | 1.70 (1.46 to 1.98) | | 1.50 (1.28 to 1.76) |
| No | 12 (33.5) | 37 (20.3) | 13.2 (-6.9 to 33.2) | 1.68 (0.86 to 3.32) | | 1.66 (0.54 to 5.08) |
| Calendar period at index date | | |  |  | |  |
| 1969-1989 | 24 (24.2) | 87 (17.8) | 6.4 (-4.0 to 16.8) | 1.46 (0.91 to 2.35) | | 1.00 (0.58 to 1.74) |
| 1990-2001 ^d^ | 103 (26.5) | 334 (17.5) | 9.0 (3.6 to 14.5) | 1.59 (1.27 to 2.01) | | 1.40 (1.10 to 1.78) |
| 2002-2011 | 94 (29.3) | 245 (15.7) | 13.6 (7.4 to 19.8) | 1.79 (1.41 to 2.29) | | 1.54 (1.19 to 2.00) |
| 2012-2023 | 34 (27.1) | 80 (13.1) | 14.0 (4.5 to 23.6) | 2.05 (1.36 to 3.10) | | 1.90 (1.23 to 2.93) |
| Educational attainment, years | | |  |  | |  |
| 0-9 | 72 (63.0) | 160 (28.2) | 34.7 (19.5 to 49.9) | 2.33 (1.73 to 3.14) | | 1.99 (1.37 to 2.91) |
| 10-12 | 91 (23.3) | 293 (15.5) | 7.8 (2.7 to 12.9) | 1.51 (1.19 to 1.93) | | 1.18 (0.88 to 1.58) |
| ≥13 | 71 (19.0) | 213 (11.7) | 7.3 (2.6 to 12.0) | 1.57 (1.19 to 2.06) | | 1.71 (1.20 to 2.44) |
| Missing | 21 (37.2) | 80 (26.6) | 10.5 (-6.4 to 27.4) | 1.57 (0.94 to 2.64) | | 0.81 (0.41 to 1.61) |
| History of metabolic-related diseases ^e,f^ | | |  |  | |  |
| Yes | 37 (44.7) | 116 (27.1) | 17.6 (2.4 to 32.9) | 1.65 (1.12 to 2.43) | | 1.43 (0.93 to 2.20) |
| No | 218 (25.6) | 630 (15.2) | 10.4 (6.8 to 14.0) | 1.71 (1.46 to 2.01) | | 1.52 (1.29 to 1.80) |
| History of autoimmune diseases ^e^ | | |  |  | |  |
| Yes | 60 (67.8) | 94 (20.8) | 47.0 (29.4 to 64.7) | 3.59 (2.55 to 5.07) | | 0.88 (0.34 to 2.29) |
| No | 195 (23.0) | 652 (15.8) | 7.2 (3.8 to 10.7) | 1.46 (1.24 to 1.72) | | 1.45 (1.23 to 1.72) |
| History of heavy alcohol consumption ^e^ |  |  |  |  | |  |
| Yes | 12 (117.1) | 18 (29.3) | 87.8 (20.2 to 155.4) | 4.02 (1.81 to 8.89) | | 3.04 (1.02 to 9.05) |
| No | 243 (26.3) | 728 (16.1) | 10.2 (6.7 to 13.7) | 1.66 (1.42 to 1.92) | | 1.46 (1.25 to 1.71) |
| History of COPD≥40 years ^e,g^ |  |  |  |  | |  |
| Yes | 3 (114.0) | 6 (33.3) | 80.7 (-51.0 to 212.4) | 3.23 (0.51 to 20.64) | | 0.75 (0.08 to 7.06) |
| No | 252 (27.0) | 740 (16.2) | 10.8 (7.3 to 14.3) | 1.70 (1.47 to 1.97) | | 1.48 (1.27 to 1.73) |
|  | **Alcohol-related AP** | | | | | |
| Age at index date |  |  |  |  | |  |
| <18 | 7 (1.7) | 21 (1.0) | 0.7 (-0.7 to 2.0) | 1.58 (0.67 to 3.75) | | 1.26 (0.48 to 3.31) |
| 18-<40 | 12 (5.5) | 56 (5.2) | 0.3 (-3.1 to 3.7) | 0.99 (0.53 to 1.86) | | 0.93 (0.45 to 1.92) |
| 40-<60 | 18 (9.3) | 75 (7.9) | 1.4 (-3.3 to 6.0) | 1.26 (0.74 to 2.13) | | 1.11 (0.62 to 1.96) |
| ≥60 | 10 (9.1) | 21 (3.9) | 5.2 (-0.7 to 11.1) | 2.36 (1.03 to 5.38) | | 2.54 (1.01 to 6.40) |
| Sex |  |  |  |  | |  |
| Female | 13 (2.2) | 49 (1.7) | 0.5 (-0.8 to 1.8) | 1.32 (0.71 to 2.45) | | 1.14 (0.58 to 2.23) |
| Male | 34 (9.9) | 124 (7.3) | 2.6 (-1.0 to 6.2) | 1.32 (0.90 to 1.95) | | 1.17 (0.77 to 1.79) |
| Born in Nordic countries ^c^ | | |  |  | |  |
| Yes | 43 (4.8) | 159 (3.6) | 1.1 (-0.4 to 2.7) | 1.31 (0.93 to 1.85) | | 1.19 (0.82 to 1.72) |
| No | 4 (11.2) | 14 (7.7) | 3.5 (-8.2 to 15.1) | 1.35 (0.43 to 4.22) | | 4.68 (0.32 to 69.4) |
| Calendar period at index date | | |  |  | |  |
| 1969-1989 | 9 (9.1) | 25 (5.1) | 4.0 (-2.3 to 10.2) | 2.08 (0.93 to 4.64) | | 1.49 (0.52 to 4.32) |
| 1990-2001 | 14 (3.6) | 65 (3.4) | 0.2 (-2.1 to 2.3) | 1.05 (0.58 to 1.90) | | 0.79 (0.40 to 1.54) |
| 2002-2011 | 18 (5.6) | 62 (4.0) | 1.6 (-1.1 to 4.4) | 1.36 (0.79 to 2.34) | | 1.39 (0.78 to 2.49) |
| 2012-2023 | 6 (4.8) | 21 (3.4) | 1.4 (-2.8 to 5.5) | 1.40 (0.56 to 3.54) | | 1.07 (0.36 to 3.17) |
| Educational attainment, years | | |  |  | |  |
| 0-9 | 12 (10.5) | 25 (4.4) | 6.1 (-0.1 to 12.3) | 2.72 (1.27 to 5.80) | | 1.97 (0.76 to 5.11) |
| 10-12 | 15 (3.8) | 78 (4.1) | -0.3 (-2.4 to 1.9) | 0.90 (0.52 to 1.57) | | 0.69 (0.37 to 1.28) |
| ≥13 | 13 (3.5) | 47 (2.6) | 0.9 (-1.1 to 2.9) | 1.24 (0.67 to 2.30) | | 2.47 (1.01 to 6.05) |
| Missing | 7 (12.4) | 23 (7.7) | 4.7 (-5.0 to 14.4) | 1.96 (0.80 to 4.80) | | 1.73 (0.45 to 6.57) |
| History of metabolic-related diseases ^e,f^ | | |  |  | |  |
| Yes | 12 (14.5) | 28 (6.5) | 8.0 (-0.6 to 16.5) | 2.14 (1.03 to 4.44) | | 2.27 (0.98 to 5.22) |
| No | 35 (4.1) | 145 (3.5) | 0.6 (-0.9 to 2.1) | 1.18 (0.81 to 1.71) | | 1.07 (0.72 to 1.60) |
| History of autoimmune diseases ^e^ | | |  |  | |  |
| Yes | 10 (11.3) | 26 (5.8) | 5.6 (-1.8 to 12.9) | 1.80 (0.85 to 3.84) | | 0.20 (0.04 to 1.10) |
| No | 37 (4.4) | 147 (3.6) | 0.8 (-0.7 to 2.3) | 1.22 (0.84 to 1.76) | | 1.15 (0.78 to 1.70) |
| History of heavy alcohol consumption ^e^ |  |  |  |  | |  |
| Yes | 19 (185.4) | 9 (14.6) | 170.7 (86.8 to 254.6) | 12.19 (4.86 to 30.61) | | 20.05 (3.97 to 101.24) |
| No | 28 (3.0) | 164 (3.6) | -0.6 (-1.9 to 0.6) | 0.83 (0.55 to 1.24) | | 0.79 (0.52 to 1.21) |
| History of COPD≥40 years ^e,g^ |  |  |  |  | |  |
| Yes | 1 (38.0) | 1 (5.6) | 32.4 (-42.8 to 107.7) | N/A | | N/A |
| No | 46 (4.9) | 172 (3.8) | 1.2 (-0.4 to 2.7) | 1.30 (0.93 to 1.81) | | 1.19 (0.84 to 1.69) |
|  | **Severe AP** | | | | | |
| Age at index date |  |  |  |  | |  |
| <18 | 11 (2.7) | 23 (1.1) | 1.5 (-0.1 to 3.2) | 2.22 (1.08 to 4.58) | | 2.12 (0.88 to 5.11) |
| 18-<40 | 13 (5.9) | 31 (2.9) | 3.0 (-0.3 to 6.4) | 2.14 (1.09 to 4.19) | | 1.50 (0.67 to 3.37) |
| 40-<60 | 43 (22.0) | 111 (11.6) | 10.3 (3.4 to 17.3) | 2.11 (1.46 to 3.06) | | 1.89 (1.28 to 2.80) |
| ≥60 | 40 (36.2) | 133 (24.8) | 11.3 (-0.7 to 23.3) | 1.32 (0.90 to 1.93) | | 1.30 (0.88 to 1.93) |
| Sex |  |  |  |  | |  |
| Female | 65 (10.9) | 149 (5.2) | 5.8 (3.0 to 8.6) | 2.05 (1.50 to 2.79) | | 1.82 (1.32 to 2.51) |
| Male | 42 (12.2) | 149 (8.8) | 3.4 (-0.5 to 7.4) | 1.48 (1.03 to 2.12) | | 1.32 (0.90 to 1.95) |
| Born in Nordic countries ^c^ | | |  |  | |  |
| Yes | 103 (11.4) | 292 (6.6) | 4.8 (2.5 to 7.1) | 1.74 (1.37 to 2.21) | | 1.55 (1.20 to 1.99) |
| No | 4 (11.1) | 6 (3.3) | 7.8 (-3.4 to 19.1) | 3.65 (0.90 to 14.78) | | N/A |
| Calendar period at index date | | |  |  | |  |
| 1969-1989 | 15 (15.0) | 45 (9.2) | 5.9 (-2.2 to 14.0) | 1.84 (0.97 to 3.46) | | 1.22 (0.60 to 2.48) |
| 1990-2001 ^d^ | 47 (12.0) | 133 (6.9) | 5.1 (1.5 to 8.7) | 1.76 (1.23 to 2.50) | | 1.59 (1.09 to 2.32) |
| 2002-2011 | 36 (11.2) | 97 (6.2) | 5.0 (1.1 to 8.8) | 1.76 (1.18 to 2.63) | | 1.62 (1.06 to 2.48) |
| 2012-2023 | 9 (7.2) | 23 (3.8) | 3.4 (-1.5 to 8.3) | 1.88 (0.86 to 4.13) | | 1.67 (0.66 to 4.24) |
| Educational attainment, years | | |  |  | |  |
| 0-9 | 35 (30.4) | 82 (14.4) | 16.0 (5.4 to 26.5) | 2.23 (1.44 to 3.46) | | 2.30 (1.32 to 4.03) |
| 10-12 | 31 (7.9) | 109 (5.8) | 2.2 (-0.8 to 5.2) | 1.39 (0.93 to 2.10) | | 0.95 (0.58 to 1.55) |
| ≥13 | 24 (6.4) | 58 (3.2) | 3.2 (0.5 to 5.9) | 1.84 (1.12 to 3.00) | | 1.63 (0.78 to 3.38) |
| Missing | 17 (29.9) | 49 (16.2) | 13.7 (-1.3 to 28.6) | 2.00 (1.09 to 3.68) | | 1.29 (0.64 to 2.60) |
| History of metabolic-related diseases ^e,f^ | | |  |  | |  |
| Yes | 18 (21.7) | 57 (13.3) | 8.4 (-2.2 to 19.0) | 1.63 (0.93 to 2.85) | | 1.41 (0.74 to 2.68) |
| No | 89 (10.4) | 241 (5.8) | 4.6 (2.3 to 6.9) | 1.81 (1.40 to 2.34) | | 1.65 (1.26 to 2.16) |
| History of autoimmune diseases ^e^ | | |  |  | |  |
| Yes | 21 (23.6) | 38 (8.4) | 15.2 (4.8 to 25.7) | 3.21 (1.82 to 5.67) | | 2.60 (0.64 to 10.58) |
| No | 86 (10.1) | 260 (6.3) | 3.8 (1.6 to 6.1) | 1.59 (1.23 to 2.06) | | 1.49 (1.14 to 1.94) |
| History of heavy alcohol consumption ^e^ |  |  |  |  | |  |
| Yes | 8 (77.5) | 5 (8.1) | 69.4 (15.2 to 123.5) | 10.94 (3.53 to 33.90) | | 13.20 (4.02 to 43.32) |
| No | 99 (10.7) | 293 (6.5) | 4.2 (2.0 to 6.4) | 1.72 (1.35 to 2.20) | | 1.54 (1.20 to 1.97) |
| History of COPD≥40 years ^e,g^ |  |  |  |  |  | |
| Yes | 2 (75.8) | 5 (27.6) | 48.2 (-59.6 to 156.0) | 3.21 (0.44 to 23.58) | | N/A |
| No | 105 (11.2) | 293 (6.4) | 4.8 (2.6 to 7.1) | 1.76 (1.39 to 2.23) | | 1.58 (1.23 to 2.02) |
| AP: acute pancreatitis; CeD: celiac disease; CI: confidence interval; COPD: chronic obstructive pulmonary disease; HR: hazard ratio; IR: incidence rate  ^a^ Model 1: conditioned on the matching variables (birth year, sex, county of residence, and calendar year).  ^b^ Model 2: model 1 and further adjusted for country of birth, educational attainment, number of healthcare visits between two years and six months before the index date, and history of autoimmune diseases.  ^c^ Nordic countries: Sweden, Denmark, Finland, Norway, and Iceland.  ^d^ The incidence rate per 100,000 person-years among reference individuals who had an index date between 1990 and 2001 was 19.3 for gallstone-related AP and 17.5 for non-gallstone-related AP. These estimates are comparable to those observed in the Swedish population during 2005 and 2013 (i.e., 16.7-19.1 for gallstone-related AP and 15.5-19.2 for non-gallstone-relate AP), the approximate calendar period when outcomes incurred among these individuals (i.e., after median follow-up of 15.5 years) [15].  ^e^ Codes for disease histories are listed in Supplementary Table S4.  ^f^ Metabolic-related diseases: hypertension, diabetes, obesity, and dyslipidemia  ^g^ Proxy for heavy smoking [11] | | | | | | |

**Table S8 Characteristics of patients with CeD and their matched reference individuals who were at risk for recurrent AP, n (%)**

|  | **Patients with CeD** | **References** |
| --- | --- | --- |
| N | 507 | 1588 |
| Age at index date, years ᵃ |  |  |
| Mean ± SD | 58.2 ± 20.9 | 57.7 ± 20.9 |
| Median (IQR) | 62.2 (43.1 - 75.5) | 61.3 (40.6 - 74.8) |
| <18 | 18 (3.6%) | 28 (1.8%) |
| 18-<40 | 93 (18.3%) | 362 (22.8%) |
| 40-<60 | 126 (24.9%) | 367 (23.1%) |
| ≥60 | 270 (53.3%) | 831 (52.3%) |
| Female | 280 (55.2%) | 928 (58.6%) |
| Born in Nordic countries ^b^ | 483 (95.3%) | 1431 (90.1%) |
| Calendar period at index date |  |  |
| 1969-1989 | 1 (0.2%) | 3 (0.2%) |
| 1990-2001 | 54 (10.7%) | 137 (8.6%) |
| 2002-2011 | 127 (25.0%) | 424 (26.7%) |
| 2012-2023 | 325 (64.1%) | 1024 (64.5%) |
| Educational attainment, years |  |  |
| 0-9 | 150 (29.6%) | 430 (27.1%) |
| 10-12 | 205 (40.4%) | 690 (43.5%) |
| ≥13 | 142 (28.0%) | 410 (25.8%) |
| Missing | 10 (2.0%) | 58 (3.7%) |
| Number of healthcare visits ^c^ |  |  |
| 0 | 150 (29.6%) | 647 (40.7%) |
| 1 | 89 (17.6%) | 262 (16.5%) |
| 2-3 | 85 (16.8%) | 275 (17.3%) |
| ≥4 | 183 (36.1%) | 404 (25.4%) |
| History of metabolic-related diseases ^d^ | 263 (51.9%) | 853 (53.7%) |
| Hypertension | 216 (42.6%) | 682 (42.9%) |
| Diabetes | 66 (13.0%) | 231 (14.5%) |
| Obesity | 26 (5.1%) | 135 (8.5%) |
| Dyslipidemia | 134 (26.4%) | 384 (24.2%) |
| History of autoimmune diseases ^d^ | 194 (38.3%) | 274 (17.3%) |
| History of heavy alcohol consumption ^d^ | 47 (9.3%) | 152 (9.6%) |
| History of COPD≥40 years ^d,e^ | 32 (6.3%) | 67 (4.2%) |
| Follow-up time for *second* AP episode, years |  |  |
| Mean ± SD | 6.6 ± 6.5 | 6.6 ± 6.5 |
| Median (IQR) | 4.5 (1.5 - 9.9) | 4.7 (1.4 - 10.1) |
| 0-0.9 | 93 (18.3%) | 342 (21.5%) |
| 1-4.9 | 171 (33.7%) | 473 (29.8%) |
| 5-9.9 | 119 (23.5%) | 369 (23.2%) |
| 10-19.9 | 95 (18.7%) | 327 (20.6%) |
| ≥20 | 29 (5.7%) | 77 (4.8%) |
| Censoring reasons during follow-up for *all recurrent* AP episodes | | |
| Incident chronic pancreatitis | 17 (3.4%) | 52 (3.3%) |
| Incident pancreatic cancer | 7 (1.4%) | 7 (0.4%) |
| Incident CeD | N/A | 7 (0.4%) |
| Emigration | 2 (0.4%) | 7 (0.4%) |
| Death | 152 (30.0%) | 415 (26.1%) |
| Study end (31 August 2024) | 315 (62.1%) | 1066 (67.1%) |
| CeD: celiac disease; COPD: chronic obstructive pulmonary disease; IQR: interquartile range: SD: standard deviation.  ᵃ 90 days after discharge from the first AP episode.  ^b^ Nordic countries: Sweden, Denmark, Finland, Norway, and Iceland. | | |
| ^c^ Between two years and six months before the date of index date.  ^d^ Codes for disease histories are listed in Table S4.  ^e^ Proxy for heavy smoking [11] | | |

**Figure S3 Alternative time intervals to define certain secondary outcomes**

**AP: acute pancreatitis**

**
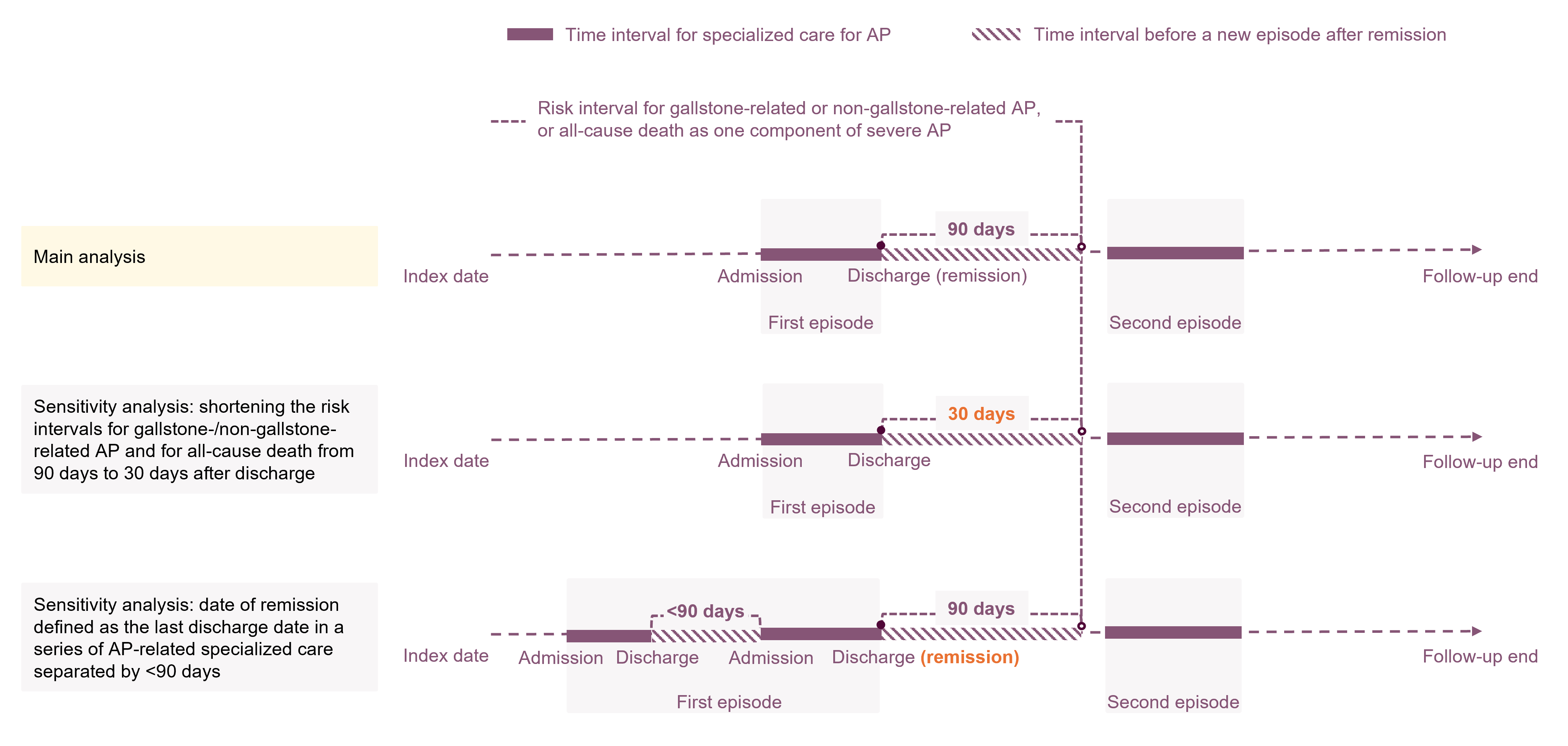
**

**Table S9 Sensitivity analyses of incident AP in patients with CeD and their matched reference individuals, by alternative time intervals to define certain secondary outcomes**

|  | No. of events (IR, per 100,000 person-years) | | IR difference (95%CI), per 100,000 person-years | HR (95%CI) ^a^ |
| --- | --- | --- | --- | --- |
|  | Patients | References |
| **Sensitivity analysis: shortening the risk intervals for gallstone-/non-gallstone-related AP and for all-cause death from 90 days to 30 days after discharge ^b^** | | | | |
| Any AP | 549 (58.7) | 1732 (37.8) | 20.9 (15.7 to 26.1) | 1.42 (1.28 to 1.58) |
| Gallstone-related AP | 253 (27.0) | 879 (19.2) | 7.8 (4.3 to 11.4) | 1.34 (1.15 to 1.55) |
| Non-gallstone-related AP | 275 (29.4) | 805 (17.6) | 11.8 (8.2 to 15.5) | 1.48 (1.27 to 1.71) |
| Alcohol-related AP | 49 (5.2) | 175 (3.8) | 1.4 (-0.2 to 3.0) | 1.24 (0.88 to 1.74) |
| Severe AP | 99 (10.6) | 281 (6.1) | 4.4 (2.2 to 6.6) | 1.56 (1.21 to 2.01) |
| Recurrent AP ^c^, among 522 patients with CeD and 1625 reference individuals who were at risk after the first episode | 143 (3724.7) | 495 (4135.1) | -410.3 (-1121.2 to 300.6) | 0.84 (0.69 to 1.03) |
| **Sensitivity analysis: date of remission defined as the last discharge date in a series of AP-related specialized care separated by <90 days ^b^** | | | | |
| Any AP | 549 (58.7) | 1732 (37.8) | 20.9 (15.7 to 26.1) | 1.42 (1.28 to 1.58) |
| Gallstone-related AP | 260 (27.8) | 898 (19.6) | 8.2 (4.6 to 11.8) | 1.34 (1.16 to 1.55) |
| Non-gallstone-related AP | 255 (27.3) | 742 (16.2) | 11.1 (7.5 to 14.6) | 1.50 (1.28 to 1.75) |
| Alcohol-related AP | 47 (5.0) | 173 (3.8) | 1.3 (-0.3 to 2.8) | 1.20 (0.85 to 1.70) |
| Severe AP | 107 (11.4) | 298 (6.5) | 4.9 (2.6 to 7.2) | 1.60 (1.25 to 2.04) |
| Recurrent AP ^c^, among 506 patients with CeD and 1577 reference individuals who were at risk after the first episode | 89 (2373.3) | 315 (2661.8) | -288.5 (-862.5 to 285.5) | 0.82 (0.64 to 1.05) |
| ^a^ Model 2: conditioned on the matching variables (birth year, sex, county of residence, and calendar year) and further adjusted for country of birth, educational attainment, number of healthcare visits between two years and six months before the index date, and history of autoimmune diseases.  ^b^ The definition for any incident AP was not affected by alternative time intervals.  ^c^ Restricted to the first three recurrence episodes for statistical power. | | | | |

**Table S****10 Characteristics of CeD patients who had a follow-up biopsy between six months and five years after diagnosis, n (%)**

|  | **Persistent villus atrophy** | **Mucosal healing** |  |
| --- | --- | --- | --- |
| N | 4099 | 11,866 |  |
| Age at index date, years ᵃ |  |  |  |
| Mean ± SD | 43.4 ± 23.7 | 30.4 ± 22.6 |  |
| Median (IQR) | 46.3 (24.6 - 63.2) | 27.7 (8.6 - 48.1) |  |
| <18 | 775 (18.9%) | 4264 (35.9%) |  |
| 18-<40 | 904 (22.1%) | 3561 (30.0%) |  |
| 40-<60 | 1218 (29.7%) | 2478 (20.9%) |  |
| ≥60 | 1202 (29.3%) | 1563 (13.2%) |  |
| Female | 2456 (59.9%) | 7736 (65.2%) |  |
| Born in Nordic countries ^b^ | 3861 (94.2%) | 11,319 (95.4%) |  |
| Calendar period at index date |  |  |  |
| 1969-1989 | 164 (4.0%) | 433 (3.6%) |  |
| 1990-2001 | 1423 (34.7%) | 3882 (32.7%) |  |
| 2002-2011 | 1376 (33.6%) | 4250 (35.8%) |  |
| 2012-2023 | 1136 (27.7%) | 3301 (27.8%) |  |
| Educational attainment, years |  |  |  |
| 0-9 | 821 (20.0%) | 1200 (10.1%) |  |
| 10-12 | 1705 (41.6%) | 4895 (41.3%) |  |
| ≥13 | 1426 (34.8%) | 5487 (46.2%) |  |
| Missing | 147 (3.6%) | 284 (2.4%) |  |
| Number of healthcare visits ^c^ |  |  |  |
| 0 | 1724 (42.1%) | 4735 (39.9%) |  |
| 1 | 784 (19.1%) | 2382 (20.1%) |  |
| 2-3 | 782 (19.1%) | 2327 (19.6%) |  |
| ≥4 | 809 (19.7%) | 2422 (20.4%) |  |
| History of metabolic-related diseases ^d^ | 725 (17.7%) | 1404 (11.8%) |  |
| Hypertension | 510 (12.4%) | 844 (7.1%) |  |
| Diabetes | 194 (4.7%) | 516 (4.3%) |  |
| Obesity | 32 (0.8%) | 102 (0.9%) |  |
| Dyslipidemia | 296 (7.2%) | 541 (4.6%) |  |
| History of autoimmune diseases ^d^ | 550 (13.4%) | 1443 (12.2%) |  |
| History of heavy alcohol consumption ^d^ | 83 (2.0%) | 158 (1.3%) |  |
| History of COPD≥40 years ^d,e^ | 31 (0.8%) | 64 (0.5%) |  |
| Follow-up time, years |  |  |  |
| Mean ± SD | 16.6 ± 9.6 | 17.6 ± 9.2 |  |
| Median (IQR) | 16.3 (8.8 - 23.9) | 17.9 (9.8 - 24.5) |  |
| 0-0.9 | 58 (1.4%) | 102 (0.9%) |  |
| 1-4.9 | 493 (12.0%) | 1165 (9.8%) |  |
| 5-9.9 | 674 (16.4%) | 1782 (15.0%) |  |
| 10-19.9 | 1364 (33.3%) | 3840 (32.4%) |  |
| ≥20 | 1510 (36.8%) | 4977 (41.9%) |  |
| CeD: celiac disease; COPD: chronic obstructive pulmonary disease; IQR: interquartile range: SD: standard deviation.  ᵃ Date of the follow-up biopsy.  ^b^ Nordic countries: Sweden, Denmark, Finland, Norway, and Iceland. | | | |
| ^c^ Between two years and six months before the date of index date.  ^d^ Codes for disease histories are listed in Table S4.  ^e^ Proxy for heavy smoking [11] | | | |

| **Table S11 Incident AP in patients with CeD who had a follow-up biopsy between six months and five years after diagnosis** | | | | | |
| --- | --- | --- | --- | --- | --- |
|  | **No. of events (IR, per 100,000 person-years)** | | **IR difference (95%CI), per 100,000 person-years** | **HR (95%CI)** | |
|  | **Persistent villus atrophy** | **Mucosal healing** |  | **Model 1 ^a^** | **Model 2 ^b^** |
| Primary outcome |  |  |  |  |  |
| Any AP | 60 (88.1) | 119 (57.1) | 31.0 (6.5 to 55.6) | 0.96 (0.69 to 1.32) | 0.92 (0.66 to 1.27) |
| Secondary outcomes | | | | | |
| Gallstone-related AP | 29 (42.5) | 54 (25.9) | 16.6 (-0.3 to 33.5) | 1.02 (0.64 to 1.64) | 1.00 (0.62 to 1.60) |
| Non-gallstone-related AP | 29 (42.6) | 56 (26.9) | 15.7 (-1.3 to 32.7) | 1.03 (0.64 to 1.64) | 0.96 (0.60 to 1.54) |
| Alcohol-related AP | 5 (7.3) | 8 (3.8) | 3.5 (-3.5 to 10.5) | 1.17 (0.36 to 3.78) | 1.17 (0.35 to 3.86) |
| Severe AP | 8 (11.7) | 21 (10.0) | 1.7 (-7.5 to 10.8) | 0.61 (0.26 to 1.42) | 0.61 (0.26 to 1.41) |
| AP: acute pancreatitis; CeD: celiac disease; CI: confidence interval; HR: hazard ratio; IR: incidence rate | | | | | |
| ^a^ Model 1: conditioned on the matching variables (birth year, sex, county of residence, and calendar year of index date).  ^b^ Model 2: model 1 and further adjusted for country of birth, educational attainment, number of healthcare visits between two years and six months before the index date, history of autoimmune diseases, and the duration between CeD diagnosis and follow-up biopsy. | | | | | |

**Table S12 Characteristics of patients with CeD and their full siblings, n (%)**

|  | **Patients with CeD** | **Full siblings** |
| --- | --- | --- |
| N | 40,256 | 69,439 |
| Age at index date, years ᵃ |  |  |
| Mean ± SD | 26.3 ± 21.4 | 28.2 ± 22.1 |
| Median (IQR) | 21.2 (7.3 - 42.9) | 24.9 (8.8 - 46.1) |
| <18 | 18,389 (45.7%) | 28,292 (40.7%) |
| 18-<40 | 10,581 (26.3%) | 18,695 (26.9%) |
| 40-<60 | 7662 (19.0%) | 15,482 (22.3%) |
| ≥60 | 3624 (9.0%) | 6970 (10.0%) |
| Female | 25,427 (63.2%) | 33,736 (48.6%) |
| Born in Nordic countries ^b^ | 39,508 (98.1%) | 67,451 (97.1%) |
| Calendar period at index date |  |  |
| 1969-1989 | 1995 (5.0%) | 3945 (5.7%) |
| 1990-2001 | 10,797 (26.8%) | 19,657 (28.3%) |
| 2002-2011 | 14,453 (35.9%) | 24,534 (35.3%) |
| 2012-2023 | 13,011 (32.3%) | 21,303 (30.7%) |
| Educational attainment, years |  |  |
| 0-9 | 3611 (9.0%) | 8388 (12.1%) |
| 10-12 | 16,275 (40.4%) | 29,032 (41.8%) |
| ≥13 | 19,356 (48.1%) | 29,759 (42.9%) |
| Missing | 1014 (2.5%) | 2260 (3.3%) |
| Number of healthcare visits ^c^ |  |  |
| 0 | 25,845 (64.2%) | 52,436 (75.5%) |
| 1 | 6131 (15.2%) | 7884 (11.4%) |
| 2-3 | 4423 (11.0%) | 5175 (7.5%) |
| ≥4 | 3857 (9.6%) | 3944 (5.7%) |
| History of metabolic-related diseases ^d^ | 4591 (11.4%) | 6004 (8.6%) |
| Hypertension | 2373 (5.9%) | 4266 (6.1%) |
| Diabetes | 2130 (5.3%) | 1635 (2.4%) |
| Obesity | 292 (0.7%) | 587 (0.8%) |
| Dyslipidemia | 1395 (3.5%) | 2548 (3.7%) |
| History of autoimmune diseases ^d^ | 4217 (10.5%) | 3016 (4.3%) |
| History of heavy alcohol consumption ^d^ | 580 (1.4%) | 1232 (1.8%) |
| History of COPD≥40 years ^d,e^ | 136 (0.3%) | 246 (0.4%) |
| Follow-up time, years |  |  |
| Mean ± SD | 17.1 ± 9.6 | 17.1 ± 9.8 |
| Median (IQR) | 16.4 (9.4 - 23.8) | 16.4 (9.1 - 24.1) |
| 0-0.9 | 366 (0.9%) | 1061 (1.5%) |
| 1-4.9 | 4095 (10.2%) | 7388 (10.6%) |
| 5-9.9 | 6550 (16.3%) | 11,167 (16.1%) |
| 10-19.9 | 14,367 (35.7%) | 23,703 (34.1%) |
| ≥20 | 14,878 (37.0%) | 26,120 (37.6%) |
| CeD: celiac disease; COPD: chronic obstructive pulmonary disease; IQR: interquartile range: SD: standard deviation.  ᵃ Date of CeD-indicative biopsy for patients with CeD and date of selection for reference individuals.  ^b^ Nordic countries: Sweden, Denmark, Finland, Norway, and Iceland. | | |
| ^c^ Between two years and six months before the date of index date.  ^d^ Codes for disease histories are listed in Table S4.  ^e^ Proxy for heavy smoking [11] | | |

**Table S13 Sensitivity analysis of incident AP in patients with CeD and their full siblings**

|  | **No. of events (IR, per 100,000 person-years)** | | **IR difference (95%CI), per 100,000 person-years** | **HR (95%CI)** | |
| --- | --- | --- | --- | --- | --- |
|  | **Patients** | **Full siblings** |  | **Model 1 ^a^** | **Model 2 ^b^** |
| Primary outcome |  |  |  |  |  |
| Any AP | 323 (46.9) | 446 (37.6) | 9.3 (3.1 to 15.5) | 1.42 (1.21 to 1.66) | 1.30 (1.10 to 1.53) |
| Secondary outcomes | | | | | |
| Gallstone-related AP | 151 (21.9) | 228 (19.2) | 2.7 (-1.6 to 7.0) | 1.24 (0.98 to 1.56) | 1.19 (0.94 to 1.50) |
| Non-gallstone-related AP | 156 (22.7) | 211 (17.8) | 4.9 (0.6 to 9.2) | 1.54 (1.23 to 1.95) | 1.32 (1.03 to 1.70) |
| Alcohol-related AP | 29 (4.2) | 49 (4.1) | 0.1 (-1.8 to 2.0) | 1.39 (0.82 to 2.35) | 1.21 (0.67 to 2.19) |
| Severe AP | 56 (8.1) | 56 (4.7) | 3.4 (0.9 to 5.9) | 2.15 (1.41 to 3.28) | 1.85 (1.16 to 2.94) |
| AP: acute pancreatitis; CeD: celiac disease; CI: confidence interval; HR: hazard ratio; IR: incidence rate | | | | | |
| ^a^ Model 1: conditioned on the matching variables (birth year, sex, county of residence, and calendar year of index date).  ^b^ Model 2: model 1 and further adjusted for country of birth, educational attainment, number of healthcare visits between two years and six months before the index date, history of autoimmune diseases, and a family identifier. | | | | | |

**Table S14 Sensitivity analysis of prior AP in patients with CeD and their matched reference individuals**

|  | **With/without prior AP ^a^** | | **OR (95%CI) ^b^** |
| --- | --- | --- | --- |
|  | **Patients** | **References** |  |
| Primary outcome |  |  |  |
| Any AP | 329/59746 | 664/296165 | 1.93 (1.67 to 2.23) |
| Secondary outcomes |  |  |  |
| Gallstone-related AP | 81/59994 | 261/296568 | 1.26 (0.97 to 1.65) |
| Non-gallstone-related AP | 218/59857 | 373/296456 | 2.20 (1.83 to 2.65) |
| Alcohol-related AP | 46/60029 | 96/296733 | 1.43 (0.93 to 2.20) |
| Severe AP | 66/60009 | 81/296748 | 2.66 (1.79 to 3.95) |
| AP: acute pancreatitis; CeD: celiac disease; CI: confidence interval; OR: odds ratio  ^a^ Excluded AP history that was preceded by chronic pancreatitis or pancreatic cancer, or gallstone-related diagnoses or procedures when investigating the history of non-gallstone-related AP or alcohol-related AP.  ^b^ Conditioned on the matching variables (birth year, sex, county of residence, and calendar year) and further adjusted for country of birth, educational attainment, number of healthcare visits between two years and six months before the index date, and history of autoimmune diseases. | | | |

| **Table S15 Sensitivity analyses of incident AP in patients with CeD and their matched reference individuals, by different exclusions and follow-up approaches** | | | | |
| --- | --- | --- | --- | --- |
|  | **No. of events (IR, per 100,000 person-years)** | | **IR difference (95%CI), per 100,000 person-years** |  |
|  | **Patients** | **References** |  | **HR (95%CI) ^a^** |
| **Restricting to individuals with available educational attainment data** | | | | |
| Any AP | 496 (56.5) | 1542 (36.0) | 20.5 (15.2 to 25.8) | 1.46 (1.32 to 1.62) |
| Gallstone-related AP | 235 (26.7) | 792 (18.4) | 8.3 (4.6 to 11.9) | 1.39 (1.20 to 1.61) |
| Non-gallstone-related AP | 234 (26.6) | 672 (15.7) | 11.0 (7.4 to 14.6) | 1.52 (1.31 to 1.78) |
| Alcohol-related AP | 40 (4.6) | 152 (3.5) | 1.0 (-0.5 to 2.5) | 1.10 (0.77 to 1.57) |
| Severe AP | 90 (10.2) | 249 (5.8) | 4.4 (2.2 to 6.7) | 1.60 (1.25 to 2.05) |
| **Excluding first year of follow-up** | | | | |
| Any AP | 509 (54.5) | 1656 (36.2) | 18.3 (13.3 to 23.3) | 1.40 (1.26 to 1.55) |
| Gallstone-related AP | 253 (27.0) | 867 (18.9) | 8.1 (4.5 to 11.7) | 1.37 (1.18 to 1.58) |
| Non-gallstone-related AP | 231 (24.7) | 718 (15.7) | 9.0 (5.6 to 12.4) | 1.40 (1.20 to 1.63) |
| Alcohol-related AP | 43 (4.6) | 171 (3.7) | 0.9 (-0.6 to 2.4) | 1.07 (0.76 to 1.51) |
| Severe AP | 99 (10.6) | 286 (6.2) | 4.3 (2.1 to 6.5) | 1.53 (1.21 to 1.93) |
| **Follow-up ended before the COVID-19 pandemic (31 December 2019)** | | | | |
| Any AP | 405 (56.4) | 1255 (35.6) | 20.8 (14.9 to 26.6) | 1.46 (1.29 to 1.64) |
| Gallstone-related AP | 178 (24.7) | 620 (17.6) | 7.2 (3.3 to 11.0) | 1.33 (1.12 to 1.59) |
| Non-gallstone-related AP | 197 (27.4) | 549 (15.6) | 11.8 (7.8 to 15.9) | 1.58 (1.32 to 1.89) |
| Alcohol-related AP | 30 (4.2) | 95 (2.7) | 1.5 (-0.1 to 3.1) | 1.36 (0.87 to 2.14) |
| Severe AP | 83 (11.5) | 218 (6.2) | 5.3 (2.7 to 8.0) | 1.73 (1.31 to 2.30) |
| **Sensitivity analyses in individuals naïve to certain medications ^b, c^** | | | | |
| **Restricting to individuals naïve to any of steroids, mesalamine, or immunosuppressants** | | | | |
| Any AP | 122 (42.6) | 501 (34.4) | 8.2 (0.0 to 16.3) | 1.16 (0.94 to 1.44) |
| Gallstone-related AP | 59 (20.6) | 271 (18.6) | 2.0 (-3.7 to 7.7) | 1.05 (0.78 to 1.41) |
| Non-gallstone-related AP | 58 (20.3) | 202 (13.9) | 6.4 (0.8 to 11.9) | 1.39 (1.01 to 1.90) |
| Alcohol-related AP | 11 (3.8) | 53 (3.6) | 0.2 (-2.3 to 2.7) | 1.15 (0.57 to 2.35) |
| Severe AP | 21 (7.3) | 80 (5.5) | 1.8 (-1.5 to 5.2) | 1.29 (0.75 to 2.21) |
| **Restricting to individuals naïve to steroids** | | | | |
| Any AP | 139 (47.5) | 516 (35.4) | 12.2 (3.7 to 20.6) | 1.23 (1.01 to 1.50) |
| Gallstone-related AP | 64 (21.9) | 277 (19.0) | 2.9 (-2.9 to 8.7) | 1.05 (0.79 to 1.40) |
| Non-gallstone-related AP | 68 (23.2) | 209 (14.3) | 8.9 (3.1 to 14.8) | 1.49 (1.11 to 2.00) |
| Alcohol-related AP | 11 (3.8) | 54 (3.7) | 0.1 (-2.4 to 2.5) | 1.08 (0.53 to 2.18) |
| Severe AP | 23 (7.9) | 82 (5.6) | 2.2 (-1.2 to 5.7) | 1.26 (0.75 to 2.12) |
| **Restricting to individuals naïve to mesalamine** | | | | |
| Any AP | 130 (44.3) | 510 (34.9) | 9.4 (1.2 to 17.6) | 1.18 (0.97 to 1.45) |
| Gallstone-related AP | 61 (20.8) | 276 (18.9) | 1.9 (-3.8 to 7.5) | 1.03 (0.77 to 1.38) |
| Non-gallstone-related AP | 63 (21.5) | 207 (14.2) | 7.3 (1.6 to 12.9) | 1.41 (1.04 to 1.91) |
| Alcohol-related AP | 13 (4.4) | 53 (3.6) | 0.8 (-1.8 to 3.4) | 1.27 (0.65 to 2.48) |
| Severe AP | 22 (7.5) | 82 (5.6) | 1.9 (-1.5 to 5.2) | 1.24 (0.74 to 2.10) |
| **Restricting to individuals naïve to immunosuppressants** | | | | |
| Any AP | 141 (47.8) | 517 (35.4) | 12.4 (4.0 to 20.9) | 1.21 (1.00 to 1.48) |
| Gallstone-related AP | 67 (22.7) | 278 (19.0) | 3.7 (-2.2 to 9.6) | 1.08 (0.82 to 1.44) |
| Non-gallstone-related AP | 66 (22.4) | 210 (14.4) | 8.0 (2.3 to 13.7) | 1.37 (1.02 to 1.83) |
| Alcohol-related AP | 12 (4.1) | 54 (3.7) | 0.4 (-2.1 to 2.9) | 1.15 (0.58 to 2.27) |
| Severe AP | 25 (8.5) | 80 (5.5) | 3.0 (-0.5 to 6.5) | 1.42 (0.86 to 2.36) |

AP: acute pancreatitis; CeD: celiac disease; CI: confidence interval; HR: hazard ratio; IR: incidence rate

^a^ Model 2: conditioned on the matching variables (birth year, sex, county of residence, and calendar year) and further adjusted for country of birth, educational attainment, number of healthcare visits between two years and six months before the index date, and history of autoimmune diseases.

^b^ Among individuals with an index date later than 1 January 2006

^c^ HRs among all CeD patients diagnosed after 1 January 2006: any AP: 1.26 (1.04 to 1.52); gallstone-related AP:1.06 (0.80 to 1.41); non-gallstone-related AP: 1.53 (1.16 to 2.03); alcohol-related AP: 1.15 (0.62 to 2.13); and severe AP: 1.36 (0.83 to 2.22).

**References**

1 Krishnan A, Teran D, Mukherjee D. Risk of incident pancreatitis in patients with celiac disease: A population-based matched retrospective cohort study. *World J Clin Cases* 2025; **13:** 112965.

2 Krishnan A, Patel R, Hadi Y, Singh S, Thakkar S. [Abstract] S95 Increased Risk of Pancreatitis, Pancreatic Cancer, and Mortality in Patients With Celiac Disease. *Official journal of the American College of Gastroenterology | ACG* 2022; **117**.

3 Alkhayyat M, Saleh MA, Abureesh M*, et al.* The Risk of Acute and Chronic Pancreatitis in Celiac Disease. *Dig Dis Sci* 2021; **66:** 2691-99.

4 Osagiede O, Lukens FJ, Wijarnpreecha K, Corral JE, Raimondo M, Kroner PT. Acute Pancreatitis in Celiac Disease: Has the Inpatient Prevalence Changed and Is It Associated With Worse Outcomes? *Pancreas* 2020; **49:** 1202-06.

5 Sadr-Azodi O, Sanders DS, Murray JA, Ludvigsson JF. Patients with celiac disease have an increased risk for pancreatitis. *Clin Gastroenterol Hepatol* 2012; **10:** 1136-42.e3.

6 Ludvigsson JF, Montgomery SM, Ekbom A. Risk of pancreatitis in 14,000 individuals with celiac disease. *Clin Gastroenterol Hepatol* 2007; **5:** 1347-53.

7 Ludvigsson JF, Brandt L, Montgomery SM, Granath F, Ekbom A. Validation study of villous atrophy and small intestinal inflammation in Swedish biopsy registers. *BMC Gastroenterol* 2009; **9:** 19.

8 Lebwohl B, Green PHR, Söderling J, Roelstraete B, Ludvigsson JF. Association Between Celiac Disease and Mortality Risk in a Swedish Population. *JAMA* 2020; **323:** 1277-85.

9 Bergman D, Hagstrom H, Capusan AJ*, et al.* Incidence of ICD-Based Diagnoses of Alcohol-Related Disorders and Diseases from Swedish Nationwide Registers and Suggestions for Coding. *Clin Epidemiol* 2020; **12:** 1433-42.

10 Miao J, Brismar K, Nyren O, Ugarph-Morawski A, Ye W. Elevated hip fracture risk in type 1 diabetic patients: a population-based cohort study in Sweden. *Diabetes Care* 2005; **28:** 2850-5.

11 Ludvigsson JF, Inghammar M, Ekberg M, Egesten A. A nationwide cohort study of the risk of chronic obstructive pulmonary disease in coeliac disease. *J Intern Med* 2012; **271:** 481-89.

12 Green PHR, Paski S, Ko CW, Rubio-Tapia A. AGA Clinical Practice Update on Management of Refractory Celiac Disease: Expert Review. *Gastroenterology* 2022; **163:** 1461-69.

13 Meczker A, Hanak L, Parniczky A*, et al.* Analysis of 1060 Cases of Drug-Induced Acute Pancreatitis. *Gastroenterology* 2020; **159:** 1958-61 e8.

14 Wintzell V, Svanstrom H, Olen O, Melbye M, Ludvigsson JF, Pasternak B. Association between use of azathioprine and risk of acute pancreatitis in children with inflammatory bowel disease: a Swedish-Danish nationwide cohort study. *Lancet Child Adolesc Health* 2019; **3:** 158-65.

15 Oskarsson V, Hosseini S, Discacciati A*, et al.* Rising incidence of acute pancreatitis in Sweden: National estimates and trends between 1990 and 2013. *United European Gastroenterol J* 2020; **8:** 472-80.
